# Changes in the Canine Plasma Lipidome after Short- and Long-Term Excess Glucocorticoid Exposure

**DOI:** 10.1038/s41598-019-42190-1

**Published:** 2019-04-12

**Authors:** Nadja S. Sieber-Ruckstuhl, Bo Burla, Susanne Spoerel, Florence Schmid, Claudio Venzin, Amaury Cazenave-Gassiot, Anne K. Bendt, Federico Torta, Markus R. Wenk, Felicitas S. Boretti

**Affiliations:** 1 Clinic for Small Animal Internal Medicine, Vetsuisse Faculty, University of Zurich, Zurich, Switzerland; 2 Singapore Lipidomics Incubator (SLING), Life Sciences Institute, National University of Singapore, Singapore, Singapore; 3 Department of Biochemistry, YLL School of Medicine, National University of Singapore, Singapore, Singapore; 4 Clinic for Small Animal Surgery, Vetsuisse Faculty, University of Zurich, Zurich, Switzerland

## Abstract

Glucocorticoids (GCs) are critical regulators of metabolic control in mammals and their aberrant function has been linked to several pathologies. GCs are widely used in human and veterinary clinical practice as potent anti-inflammatory and immune suppressive agents. Dyslipidaemia is a frequently observed consequence of GC treatment, typified by increased lipolysis, lipid mobilization, liponeogenesis, and adipogenesis. Dogs with excess GC show hyperlipidaemia, hypertension, and a higher risk of developing type 2 diabetes mellitus, but the risk of developing atherosclerotic lesions is low as compared to humans. This study aimed to examine alterations in the canine plasma lipidome in a model of experimentally induced short-term and long-term GC excess. Both treatments led to significant plasma lipidome alterations, which were more pronounced after long-term excess steroid exposure. In particular, monohexosylceramides, phosphatidylinositols, ether phosphatidylcholines, acyl phosphatidylcholines, triacylglycerols and sphingosine 1-phosphates showed significant changes. The present study highlights the hitherto unknown effects of GCs on lipid metabolism, which will be important in the further elucidation of the role and function of GCs as drugs and in metabolic and cardiovascular diseases.

## Introduction

Glucocorticoids (GCs) are highly effective anti-inflammatory and immunosuppressant drugs, commonly used to treat acute and chronic inflammatory and immune-mediated diseases. Besides their positive therapeutic effects, GCs also have many metabolic side effects, which are typically more severe after high doses and chronic applications. These adverse effects include hypertension, thin and fragile skin, wound healing disturbances, osteoporosis, muscle atrophy, weight gain, steroid diabetes, and venous thromboembolism^1–3^. The clinical signs of chronic GC applications are summarised in iatrogenic Cushing’s syndrome (CS), where patients present with increased morbidity and mortality primarily due to cardiovascular, thrombotic, and metabolic complications^4–6^. CS can also arise from endogenous cortisol overproduction but both forms lead to indistinguishable clinical signs. Dyslipidaemia is a frequent feature in patients with CS, resulting from a GC-induced increase in lipolysis, lipid mobilization, liponeogenesis, and adipogenesis. Hypercholesterolemia, increased triglyceride levels, and alterations in HDL cholesterol and LDL/HDL ratios are also typically observed^7–10^. An aggressive lipid-lowering management is recommended to lower the cardiovascular disease (CVD) risk in these patients^11^.

Dogs have been used as models to study metabolic obesity, diabetes, dyslipidaemia and response to pharmacologic interventions^12^. In dogs, treatment with GCs causes many of the side effects seen in humans, and extended GC therapy can induce iatrogenic CS. Dogs with endogenous or iatrogenic CS show hyperlipidaemia, hypertension, and have a higher risk of developing type 2 diabetes mellitus^13,14^. However, CS in dogs does not appear to increase the risk of developing atherosclerotic lesions^15^. This may be explained by difference in their lipid metabolism: unlike humans, dogs have very low cholesteryl ester transfer protein (CETP) activity, an enzyme that mediates the transport of triglycerides (TG) from LDL and VLDL to HDL_2_ and of cholesteryl esters from HDL_2_ to VLDL and LDL^13,16,17^. Dogs carry most of their plasma cholesterol on HDL particles, resulting in an athero-protective profile, as high HDL-cholesterol levels are associated with a low risk of CVD^13,16,17^.

In recent years, lipidomics has emerged as a new field involving the large-scale study of novel lipids as functional elements and markers of disease^18,19^. Although little is known about the plasma lipidome composition in dogs^17,20^, a recent study suggests that dogs show the highest overall similarity to patients with dyslipidaemia in terms of their lipid profiles and responses to the statin, simvastatin^17^. Therefore, dogs may represent a relevant model to evaluate the effects of GCs on lipid metabolism. Thus, the aim of this study was to examine changes in the canine plasma lipidome after short-term and long-term exposure to excess GC. In the first experiment, healthy Beagle dogs received a long-term treatment with tetracosactide, a synthetic peptide, comprising the first 24 amino acids of the adrenocorticotropic hormone (ACTH), which induced chronic endogenous cortisol secretion (experimentally induced CS model)^21^. In the second experiment, dogs received a short-term treatment of a high-dose of prednisolone to study the acute effects of GC use.

## Results

### Clinical chemistry and phenotypes

**Figure 1:**
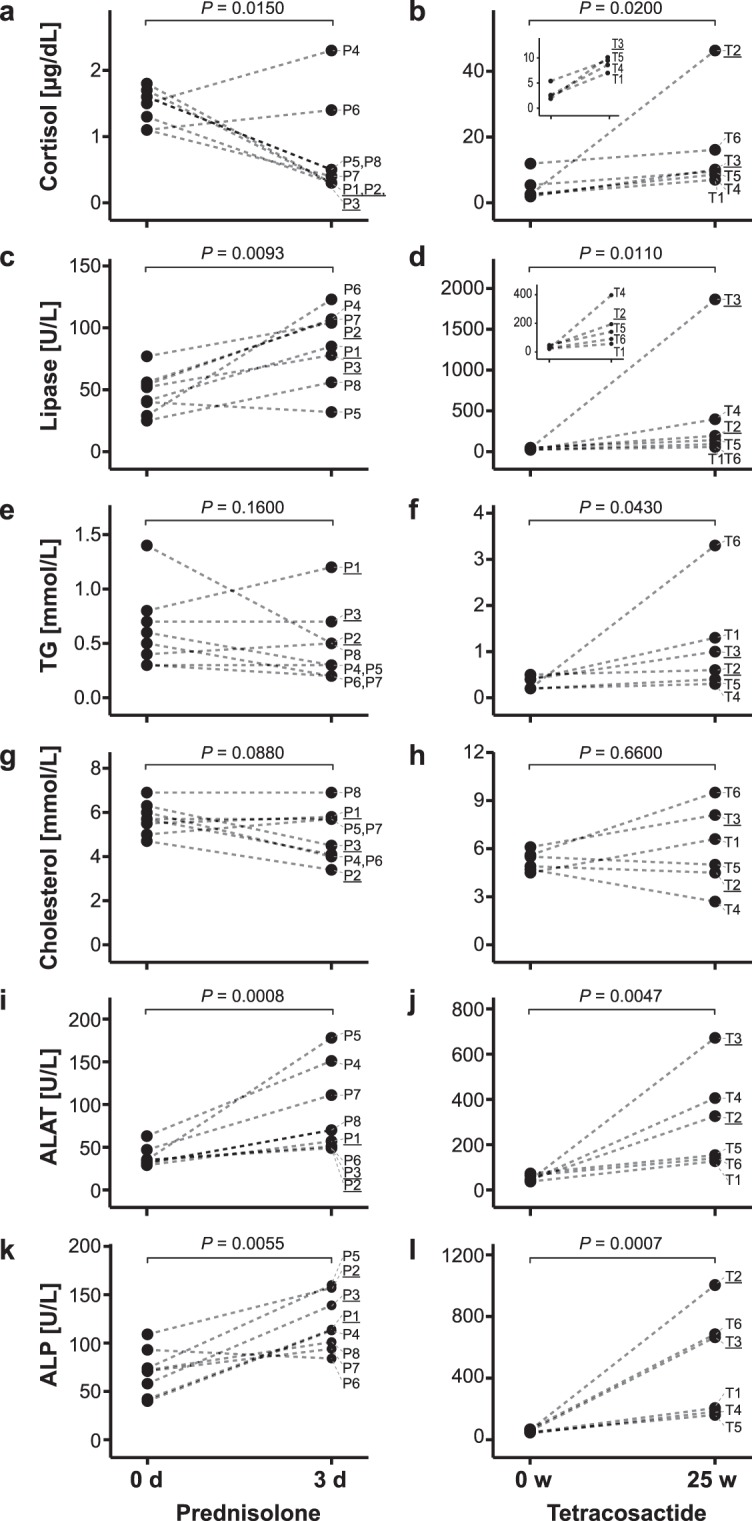
Changes in clinical plasma markers of lipid metabolism. Levels for each dog before (0 d and 0 w, respectively) and after (3 d and 25 w, respectively) treatment with short-term prednisolone (left) or long-term tetracosactide (right). Values from the same dog, before and after treatment, are connected with dotted lines. The *P* values of paired two-tailed *t* tests comparing log-transformed values before and after treatment are indicated at the top of each panel. Small inserts illustrate data at a magnified scale. ALAT, alanine aminotransferase activity; ALP alkaline phosphatase activity; TG triglycerides.

Selected clinical chemistry parameters were measured before and after treatment to monitor the efficacy of prednisolone and tetracosactide treatments and the general health of the animals during the experiments (Fig. 1 and Supplementary Table S1). Short-term prednisolone treatment led to a decrease in serum cortisol levels in six of eight dogs (median of all dogs: −3.2-fold; Fig. 1a). However, dogs P4 and P6 had increased cortisol levels (1.4-fold). By comparison, long-term tetracosactide treatment significantly increased plasma cortisol levels (median: 3.1-fold; Fig. 1b). Plasma lipase activities significantly increased after both treatments, with more relevant changes observed for dogs receiving tetracosactide (median prednisolone: 1.9-fold, tetracosactide: 3.9-fold; Fig. 1c,d). Dog T3 showed a considerably higher lipase activity compared to the others. Plasma triglycerides (TG) were significantly increased after long-term tetracosactide treatment (median: 2.2-fold) but not after short-term prednisolone treatment (Fig. 1e,f). Dog T6 showed a higher increase in TG levels compared to the other dogs after tetracosactide treatment. Neither treatment had a significant effect on total cholesterol levels (Fig. 1g,h). Alanine aminotransferase and alkaline phosphatase activities were significantly increased in all dogs after both treatments (median prednisolone: 2.2 and 1.8-fold, respectively, tetracosactide: 5.3 and 7.3-fold, respectively; Fig. 1i-l).

To further test the efficacy of long-term tetracosactide treatment, we performed low-dose dexamethasone suppression (LDDS) and ACTH stimulation tests^21,22^. We found that all dogs were positive for both tests (see Supplementary Table S1). Furthermore, long-term tetracosactide treatment resulted in a significant body weight loss in all dogs (range: −2.1 to −4.4 kg, median: −3.0 kg, *P* = 0.0004; see Supplementary Table S1). The body weights were not recorded after short-term prednisolone treatment, as no relevant changes were expected after such a short treatment. All dogs showed polyuria, polydipsia and polyphagia after prednisolone treatment. After long-term tetracosactide, all but one (T6) dog showed polyuria and polydipsia. Muscle wasting was detected in all tetracosactide-treated dogs, except for dogs T1 and T6, and increased central obesity was observed for dogs T2 and T3 (Supplementary Table S1).

### PCA and clustering analyses of the full lipidomic dataset

**Figure 2:**
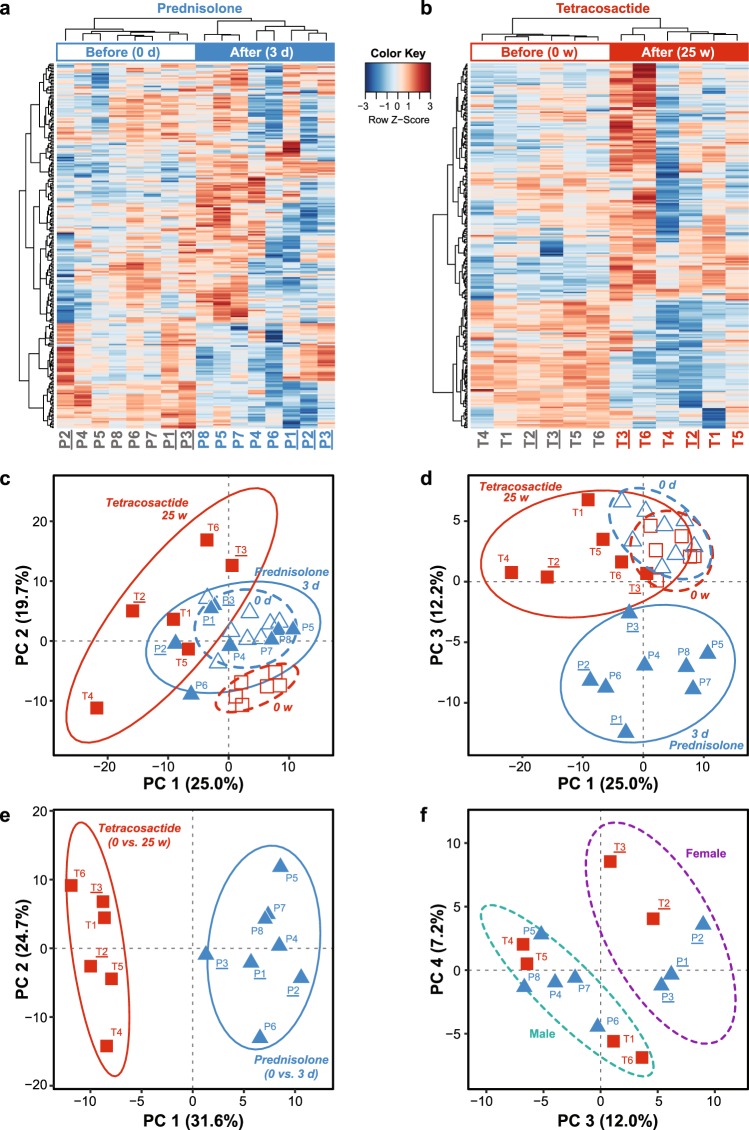
Principal component (PCA) and clustering analyses of the plasma lipidomic datasets before and after treatments. All plots are based on concentrations of all quantified lipid species in all plasma samples before and after short-term (3 days) prednisolone (dogs P1-P8) or long-term (25 weeks) tetracosactide (dogs T1-T6) treatments. Female dogs have underlined IDs. **(a,b)** Heatmaps showing the full datasets with abundance from all quantified lipid species in the plasma samples before (empty bar) and after (filled bar) treatment with short-term prednisolone (A, blue) or long-term tetracosactide (B, red). Colour scale represents normalized and centered lipid concentrations per lipid species (in rows). Dog IDs in grey font indicate before, and blue and red, after prednisolone and tetracosactide treatment, respectively. **(c,d)** PCA showing the specific effects of long-term tetracosactide (red squares) treatment in principal components 1 (PC 1) and of short-term prednisolone treatment (blue triangles) in principal components 3 (PC 3). Samples taken before treatment are indicated with empty symbols and samples after treatment with filled symbols. **(e,f)** PCA of log2-fold changes before and after short-term prednisolone (dogs P1-P8, blue triangles) and long-term tetracosactide (dogs T1-T6; red squares) treatments, respectively. PC 1 vs. PC 2 highlights distinct plasma lipidome changes between the two treatments **(e)**, whereas PC 3 vs. PC 4 indicates sex-specific effects of the treatments on the plasma lipidome, separating female and male dogs into two clusters ((**e**); purple and grey ellipses, respectively).

Hierarchical clustering based on the abundance of all measured lipid species (Supplementary Table S2) could clearly discriminate plasma samples taken before and after treatment with prednisolone and tetracosactide (Fig. 2a,b). Principal component analysis (PCA) based on the abundance of all measured lipid species separated the samples taken before and after treatments, and showed the distinct effects of long-term tetracosactide treatment, mostly evidenced by principle components PC 1 (Fig. 2c,d) and of short-term prednisolone treatment, evidenced by PC 3 (Fig. 2d). PCA plots obtained from fold changes of all quantified lipid species (Supplementary Table S3) showed a separation between prednisolone- and tetracosactide-treated dogs, mostly evidenced by PC 1 (Fig. 2e) and a separation between the sexes, evidenced by PC 3 and PC 4 (Fig. 2f).

### Ceramides (Cer), Monohexosylceramides (Hex1Cer), Dihexosylceramides (Hex2Cer) and GM3

**Figure 3:**
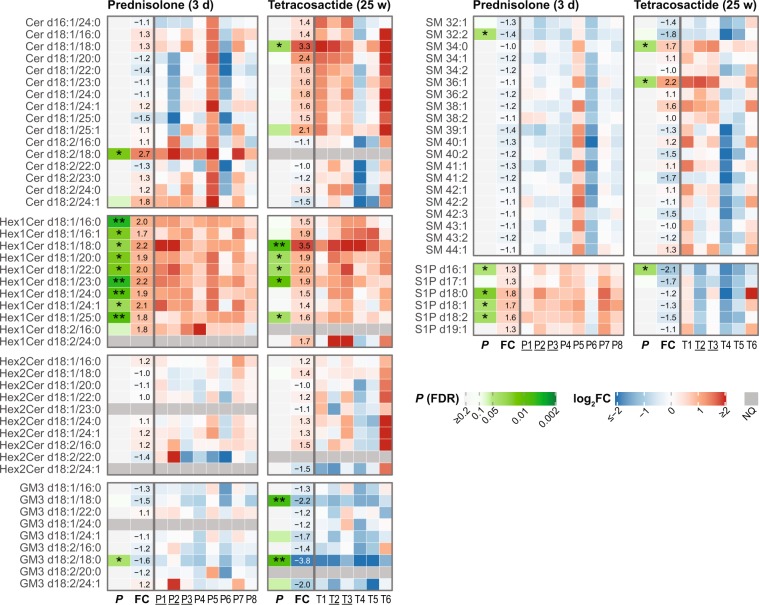
Individual changes in plasma levels of measured plasma sphingolipid species after short-term (3 days) prednisolone (left panels) and long-term (25 weeks) tetracosactide (right panels) treatments. The first column (*P*; green white colour scale) indicates the FDR-adjusted *P* values of the paired *t*-test comparing log2-transformed concentrations before and after treatment (*P ≤ 0.05, **P ≤ 0.01). The second column (FC) indicates average fold changes after vs. before treatment (color scale corresponds to log2-fold changes (log2FC), the value in the fields to fold changes). Subsequent columns indicate log2-fold changes for the individual dogs (P1-P8 for prednisolone, T1-T6 for tetracosactide; female dogs are underlined) after vs. before treatment. |FC| > 4 are capped at the colour scale with the maximum red and blue colour. Dark grey rows (NQ) indicate lipid species that were not detectable or did not meet the quality control criteria in the corresponding treatment group.

After short-term prednisolone treatment, only Cer d18:2/18:0 was significantly increased among the 16 measured ceramides (2.7-fold; Fig. 3), whereas tetracosactide induced an increase in Cer d18:1/18:0 (3.3-fold). As an exception, dog T4 showed decreased levels in almost all other Cer species. Excluding dog T4, there was an increasing trend in the levels of Cer d18:1 Cer species, even though most of these changes were not statistically significant (see Supplementary Fig. S4 and Supplementary Table S5). Nine of the ten quantified monohexosylceramides (Hex1Cer) were significantly increased after prednisolone treatment (1.7 to 2.2-fold; Fig. 3). After tetracosactide treatment, five Hex1Cer species were significantly increased (1.6 to 3.5-fold; Fig. 3). None of the quantified Hex2Cer species were significantly changed after either treatment (Fig. 3). Monosialodihexosylgangliosides (GM3) were the only gangliosides measured in this study. After prednisolone, GM3 d18:2/18:0 was significantly decreased (1.6-fold), whereas, after tetracosactide treatment, GM3 d18:1/18:0 and GM3 d18:2/18:0 were significantly decreased (2.2 and 3.8-fold; Fig. 3).

### Sphingomyelins (SM)

Prednisolone did not induce any significant changes in the levels of 20 measured SM species, except for SM 32:2 (Fig. 3). However, dog P5 showed higher, and dog P6, lower, levels in almost all SM species compared to the average trend in the prednisolone group. Long-term tetracosactide treatment resulted in significant increases in SM 34:0 and SM 36:1 (1.7 and 2.2-fold). In particular, dogs T4 and T5 showed decreased levels in all other SM species, whereas the remaining dogs showed variable, non-significant changes.

### Sphingosine 1-phosphate (S1P)

The major plasma S1P species, S1P d16:1, S1P d18:0, S1P d18:1 and S1P d18:2, were all significantly increased after short-term prednisolone treatment (1.3 to 1.8-fold; Fig. 3). However, in dog P6, the levels of all S1P species were decreased or only slightly increased. The same dog also showed decreased levels of most of the measured ceramides and sphingomyelin species, all belonging to the sphingolipid pathway. After long-term tetracosactide treatment, only S1P d16:1 was significantly decreased (2.1-fold) in all dogs; none of the other S1P species showed a consistent change (Fig. 3).

### Ether-Lysophosphatidylcholines (LPC-O), Ether-PC (PC-O), plasmalogen PCs (PC-P), Ether- phosphatidylethanolamines (PE-O) and plasmalogen-PE (PE-P)

**Figure 4:**
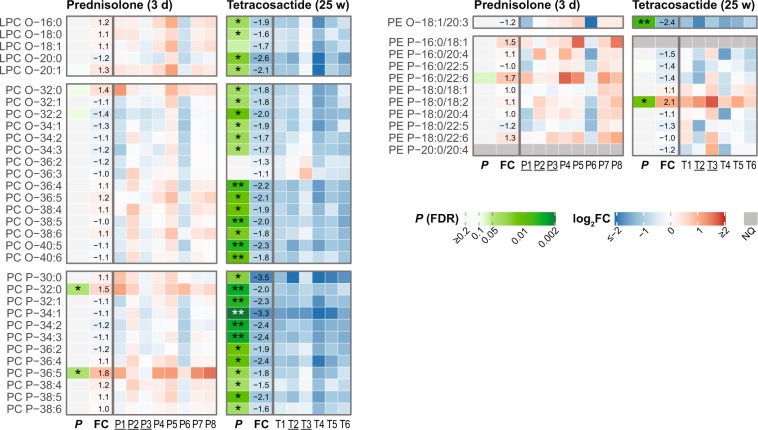
Individual changes in plasma levels of measured plasma ether-linked and plasmalogen phospholipid species after short-term (3 days) prednisolone (left panels) and long-term (25 weeks) tetracosactide (right panels) treatments. See Fig. 3 for further descriptions.

After short-term prednisolone treatment, none of the measured five LPC-O, 15 PC-O, one PE-O and 9 PE-P species were changed, and only two of the 12 PC-Ps were significantly increased (Fig. 4). On the other hand, long-term tetracosactide treatment resulted in a significant decrease in four of the five LPC-O species, 13 of the 15 PC-O species and all 12 PC-P species (−1.5 to −3.5-fold; Fig. 4). PE-O 18:1/20:3 was also significantly decreased (2.4-fold), whereas among all nine PE-P species, PE-P 18:0/18:2 was significantly increased (2.1-fold) and the others were unchanged (Fig. 4).

### Lysophosphatidylcholines (LPC)

**Figure 5:**
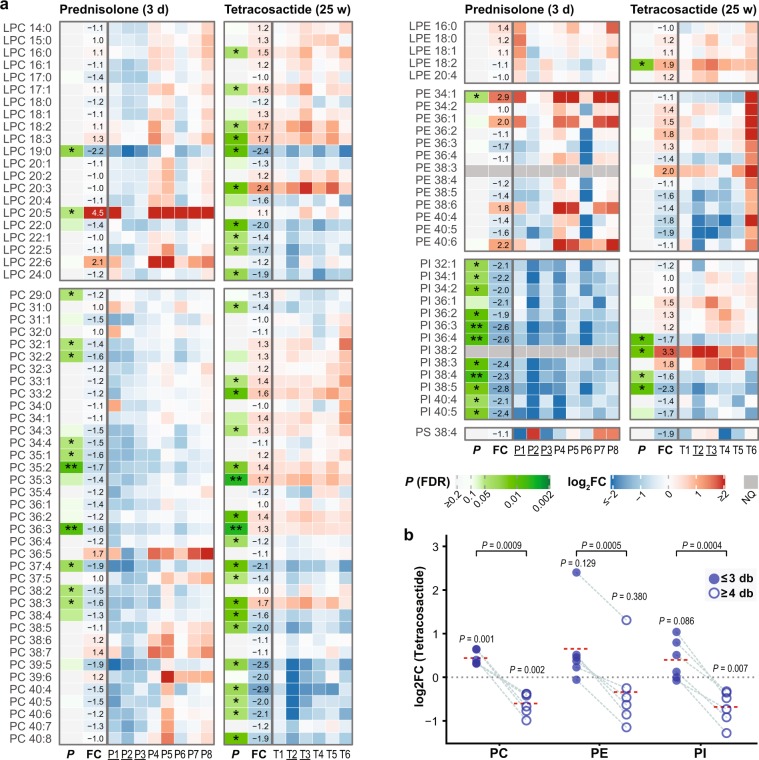
Individual changes in plasma levels of measured plasma acyl phospholipids after short-term (3 days) prednisolone (left panels) and long-term (25 weeks) tetracosactide (right panels) treatments. **(a)** Changes in individual acyl phospholipid species. See Fig. 3 for further descriptions. **(b)** Changes in total levels of PC, PE and PI lipid species with either 0 to 3 (≤3, filled circles) or 4 to 8 (≥4, open circles) fatty acid double bonds after long-term tetracosactide treatment. Values originating from the same dog are linked by grey dashed lines; the red dotted lines represent averages. *P* values were calculated from paired two-tailed *t* tests comparing log-transformed total abundances before and after treatment (shown above the points), and comparing log2FC of lipids with ≤3 and ≥4 double bonds, respectively (shown on the top).

Among the 21 measured LPC species, LPC 19:0 was significantly decreased (2.2-fold) and LPC 20:5 significantly increased (4.5-fold) after short-term prednisolone treatment (Fig. 5a). However, in dogs P2 and P3, LPC 20:5 and also LPC 22:6 were unchanged or decreased, while in other six dogs they were strongly increased (5.3 to 12.8-fold, and 1.5 to 6.0-fold, respectively; see Supplementary Table S3). After long-term tetracosactide treatment, there was a significant decrease in the levels of five out of 11 LPC species bearing fatty acid chain-lengths longer than 18 carbons (Fig. 5a). In contrast, there was a significant increase in LPC 20:3 (2.4-fold) and in several LPCs bearing chain lengths shorter than 19 carbons, i.e. LPC 16:0, 17:1, LPC 18:2 and LPC 18:3 (1.5 to 1.7-fold).

### Phosphatidylcholines (PC)

Short-term prednisolone treatment resulted in a significant decrease in 10 of the 37 measured PC species (Fig. 5a). In comparison, long-term tetracosactide treatment led to a significant decrease in the concentration of 10 PC species containing ≥4 double bonds, and in a significant increase in the concentration of eight PC species containing ≤3 double bonds (Fig. 5a,b). The ratio between the total concentration of PC species containing ≥4 and species with ≤3 double bonds was also significantly decreased (Fig. 5b).

### Lyso-Phosphatidylethanolamines (LPE) and Phosphatidylethanolamines (PE)

Prednisolone treatment had no effect on any of the five measured LPE species, whereas LPE 18:2 was increased after tetracosactide treatment (Fig. 5a). Of the 12 PE species tested, prednisolone only significantly increased the levels of PE 34:1 (Fig. 5a). Tetracosactide had no significant effect on the individual and total levels of the 13 measured PE species (Fig. 5a,b); however, there was a considerable increase in all measured PE species in samples from dog T6. Excluding dog T6, a possible trend in decreased levels of individual PE species with ≥4 double bonds, and a trend in increased levels of PE species with ≤3 double bonds can be seen (Supplementary Fig. S4 and Supplementary Table S6). The ratio between the total level of PE species containing ≥4 and species with ≤3 double bonds was significantly decreased, independently of whether dog T6 was excluded or not (Fig. 5b and Supplementary Fig. S4). When excluding dog T6, a significant decrease in the total level of PE species with ≥4 double bonds and a significant increase in the total level of PE species with ≤3 double bonds was found (Supplementary Fig. S4).

### Phosphatidylinositols (PI)

Short-term prednisolone treatment led to a significant decrease in 11 of the 12 quantified PI species (−1.9 to −2.8-fold; Fig. 5a). PI 38:2 was not quantified in short-term prednisolone treatment samples, as in this group it did not meet the analytical QC criteria. After tetracosactide treatment, a significant decrease in the levels of three molecular species (PI 36:4, PI 38:4, PI 38:5; 1.6 to 2.3-fold) and in the total levels of PIs with ≥4 double bonds was observed. PI 38:2 was significantly (3.3-fold) and the total levels of PI species with ≤3 double bonds were non-significantly increased (Fig. 5a,b). The ratio between the total concentration of PI species containing ≥4 and ≤3 double bonds was also significantly decreased (Fig. 5b).

### Phosphatidylserines (PS)

The only quantifiable PS species (PS 38:4) did not significantly change after the treatments (Fig. 5a).

### Diacylglycerols (DG) and triacylglycerols (TG)

**Figure 6:**
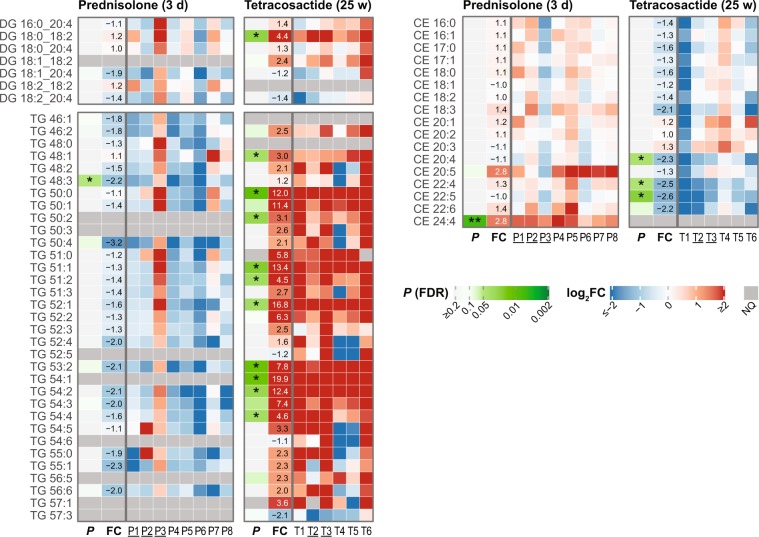
Individual changes in plasma levels of measured plasma glycerolipids and cholesteryl esters after short-term (3 days) prednisolone (left panels) and long-term (25 weeks) tetracosactide (right panels) treatments. See Fig. 3 for further descriptions.

Prednisolone treatment led to a statistically significant decrease in only one (TG 48:3) of the 25 quantified TG species (Fig. 6). None of the six measured DGs were significantly changed. However, one dog (P3) showed increased levels of DG and TG species following prednisolone treatment (Fig. 6). When P3 was excluded, a significant decrease in one DG and nine TG species was observed (see Supplementary Fig. S4 and Table S7). Pronounced changes were observed after long-term tetracosactide treatment, with a significant increase in 10 of the 31 measured TGs (3.0 to 19.9-fold); other species showed non-significant trends in increased levels (Fig. 6). For some TG species, one or two dogs (T4 and T5) had decreased, while the other dogs had increased levels. Among the six measured DGs species, only DG 18:0_18:2 was significantly elevated (4.4-fold), however a trend in increased levels in three additional DG species was observed (Fig. 6).

### Cholesteryl esters (CE)

Short-term prednisolone exposure did not lead to any significant changes in the 17 quantified CE species, except of CE 24:4, which was increased (2.8-fold; Fig. 6). Tetracosactide induced a significant decrease in the levels of CE 20:4, CE 22:4 and CE 22:5 (2.3 to 2.6-fold; Fig. 6).

## Discussion

Dyslipidemia is a common consequence of exposure to excess endogenous or exogenous GC. Dogs may represent a relevant model to study dyslipidemia and to evaluate the effects of GCs on lipid metabolism^17,23^. Here, we sought to examine how short-term and long-term GC excess alters the canine plasma lipidome.

Short-term prednisolone treatment resulted in typical clinical signs (polyuria, polydipsia and polyphagia) and increased serum lipase activity. The decrease in serum cortisol concentrations during prednisolone treatment is expected as a result of the negative feedback of the exogenously administered GC^22^. In two dogs, however, serum cortisol levels were slightly increased, which may be ascribed to the cross-reactivity of the assay with prednisolone that was still present in the serum^22^. The weight loss, the increased serum cortisol, lipase activity, and TG levels, and the positive adrenal function tests of the long-term tetracosactide-treated dogs are consistent with hypercortisolism and the symptoms of CS. The increased serum alanine aminotransferase and alkaline phosphatase activities after both treatments are likely the result of GC-induced hepatopathies and dog-specific GC-induced over-expression of alkaline phosphatase isoforms^24,25^. Considering that a substantial percentage of circulating lipids are transported by lipoproteins synthesised in the liver, it is plausible that hepatopathy could contribute to plasma lipidome changes.

The treatments with short-term prednisolone and long-term tetracosactide had distinct effects on the dog plasma lipidomes (Fig. 2). Of note, there were variabilities among individual dogs in the responses of the clinical chemistry parameters and the plasma lipidome to the treatments, which cannot be attributed to the specific dog. Two limitations in this study were the small sample sizes of the experimental groups and the heterogeneity in age of the individual dogs. Despite these age differences, the body weights of the dogs were comparable and were indicative of fully grown dogs in all groups. The lipid profiles diverged more between individual dogs after the treatments, indicating individuality in the response to the treatments (Fig. 2c,d). Biological and experimental factors, such as differences in resorption, pharmacokinetics, adrenal response, and GC sensitivity may explain such variabilities and have been reported previously for both humans and dogs^26,27^. Biological variability is expected within dog cohorts, which are usually more heterogeneous than, for example, mice cohorts. A sex-specific response to GC, affecting plasma lipids, have been observed in humans^28–30^. Each treatment group in our study included about two-thirds male and one-third female dogs. We observed sex-specific effects on the plasma lipidome in both treatments; however, these effects were weaker than the effects of the treatment (Fig. 2e,f). Given the already small sample size, we have not further investigated which lipids contribute to these sex-specific differences. In summary, the differences in overall plasma lipid profiles induced by the treatments were larger than the effects of gender, age, weight, and other possible confounding factors.

The most substantial changes in a lipid class occurring in both short-term prednisolone and long-term tetracosactide exposures were found for Hex1Cer levels (Fig. 3). The increased synthesis of Hex1Cer species seen in our study could be attributed to an increase in their precursor ceramide or could be the result of an altered catabolism of complex glycosphingolipids. Interestingly, no significant changes to Hex2Cer were found, which derives from Hex1Cer; yet, a trend towards increased ceramide synthesis was present in response to long-term tetracosactide treatment (Fig. 3). This corresponds to findings in mice, where a seven days exposure to the GC dexamethasone resulted in an increased synthesis of specific plasma ceramides^31^. However, Cer levels did not change after short-term prednisolone treatment, which may have been too short to induce the ceramide production. After long-term tetracosactide treatment, only one ceramide species was significantly elevated, however, other d18:1 ceramides tended to be elevated.

A biologically important class of sphingolipids is S1P, which is implicated in various biological processes, including immune reactions, vascular integrity, blood coagulation, tissue growth, apoptosis and human diseases^32–34^. After short-term prednisolone treatment, we measured an increase in the four most abundant S1P molecular species, which are also the most abundant in human plasma^35^. Vettorazzi *et al*. showed that dexamethasone treatment led to a significant increase in plasma S1P levels in mice, which they ascribed to an induction in the expression of sphingosine kinase 1 (SphK1), an enzyme that phosphorylates sphingosine to S1P^36^. It is possible that the short-term prednisolone treatment in our study led to an increased synthesis of S1P, which might be implicated in the regulation of inflammatory processes^37^. Long-term tetracosactide treatment significantly decreased the levels of the low-abundant S1P d16:1. No data is available yet in the literature on the functions of S1P d16:1, probably because only recently it was possible to quantify this species in plasma^35^. However, it has been shown that sphingolipids containing the d16:0 and d16:1 sphingoid bases have, compared with the more abundant d18-containing sphingolipids, different biophysical and biological properties and seem to be important in cardiac functions^38,39^.

The most relevant change induced by tetracosactide treatment was measured for ether-linked PCs. Long-term tetracosactide exposure led to a significant reduction in the plasma levels of 29 of the 32 measured ether-linked PC species, while short-term prednisolone did not affect this lipid class (Fig. 4). Ether-linked PCs are synthesised via a distinct pathway compared to acyl PCs^40^. LPC-Os (also named Lyso-PAF) are the precursors for platelet-activating factors (PAF). Lyso-PAF and PAF are signalling molecules implicated in platelet reactivity, endothelial function, and various inflammatory processes^41^. The decreased levels of all measured Lyso-PAF species after long-term tetracosactide treatment—which might consequently reflect altered levels of PAF—may therefore influence inflammatory processes. PC-Ps are normally enriched in the polyunsaturated fatty acids (PUFAs) arachidonic acid (AA) and docosahexaenoic acid (DHA), which are precursors of eicosanoids and related compounds^40^. Reduced circulating PC-P levels may also affect the protective function of lipoproteins, as ether- and plasmalogen phospholipids can act as scavengers of reactive oxygen species^40^. In contrast to our results, most of the ether-linked PC species were unchanged in human CS patients compared to healthy individuals^42^. This may indicate either a difference between dogs and humans in the effects of GCs on this lipid class, and/or a difference in tetracosactide-induced hypercortisolism in contrast to endogenous hypercortisolism in humans. The significance and biological role of these changes in ether-linked PCs in dogs after long-term tetracosactide will have to be better investigated in the future. PE-Ps were not affected by the tetracosactide treatment, suggesting different functions or regulations of this lipid class compared to ether-linked PCs.

Another distinct and consistent effect of short-term prednisolone treatment—besides the effects on Hex1Cer—concerns the PI species, which were significantly decreased (Fig. 5a). PIs are sources of arachidonic acid and precursors of phosphatidylinositol phosphates (PIPs), important signalling molecules in inflammatory processes^43,44^. Phosphoinositide 3-kinases (PI3K) catalyse the synthesis of PIP from PI and interact with GCs during the innate immune response^45^. It will be interesting to further investigate the mechanism and physiological implication of these significant changes in PI levels.

After long-term tetracosactide treatment, a significantly decreased ratio between PCs, PEs and PIs with ≥4 and species with ≤3 double bonds was observed (Fig. 5a,b). This difference is mainly caused by a decrease in species with ≥4 and increases in those with ≤3 double bonds (Fig. 5a,b). Others have shown that steroids and ACTH can inhibit Δ5- and Δ6-desaturases in the liver and adrenal glands of rats, resulting in reduced synthesis of 18:3n-6, 18:4n-3 and 20:4n-6 fatty acids^46^. GCs may therefore reduce the synthesis of PUFA-containing phospholipids and hence lower the availability of the precursor pool for eicosanoids, important factors in inflammatory processes^47,48^. In human patients with CS, plasma levels of PC 38:3, PC 38:4, and PC 40:4 were all reduced as compared with healthy subjects^42^. In our study, however, PC 38:3 was significantly increased. In human plasma, PC 38:3 species mainly consists of PC 18:0/20:3^49,50^. Interestingly, LPC 20:3 was also strongly increased. A more detailed analysis of the fatty acid components will be helpful in future to better understand the effects of excess GC on the fatty acid metabolism.

After short-term prednisolone treatment, approximately one-fourth of the measured PC species were significantly decreased, whereby no effects based on acyl chain-length or saturation could be identified (Fig. 5a). These findings overlap with published data on reduced plasma PC levels after a single-dose of dexamethasone in humans^51^, which presumably result from GC-induced lipolysis.

In agreement with elevated plasma TG levels previously reported in humans and dogs with CS^22,52^, we observed significantly elevated levels of various TG species after long-term tetracosactide treatment (Fig. 6). These increases were reflected in the total TG levels reported by clinical chemistry measurements (Fig. 1f). However, in contrast to the typically elevated total cholesterol levels in dogs and human with CS^13,16,51^, we did not observe any significant increases in serum total cholesterol and plasma CE levels (Figs 1h and 6). Three PUFA-containing CE species were also significantly decreased, which may reflect the same observation made for PUFA-containing acyl-glycerophospholipids.

The short-term prednisolone treatment led to a trend in reduced levels of many TG species (Fig. 6 and Supplementary Fig. S4). This observation matches previous findings in dogs and humans, where a single dose of dexamethasone resulted in decreased plasma TG levels^51,53^. However, our TG lipidomic results are not reflected by the total TG levels measured by clinical chemistry (Fig. 1e). This discrepancy may be explained by the overall small effects on total TGs and significant differences in the levels of specific TG species only. CE and total cholesterol levels did not change, in agreement with previously reported effects of a single dose of dexamethasone in human and dogs^51,53^.

Our study revealed specific effects of both treatments on the plasma lipidome that were consistently observed across all individuals: (i) the increase in Hex1Cer after both short-term prednisolone and long-term tetracosactide treatments; (ii) the decrease in PIs after short-term prednisolone treatment; and after long-term tetracosactide, (iii) the decrease in ether-phospholipids, (iv) the increase of TGs and, (v) the decrease in the ratio of acyl phospholipids with 4 or more unsaturations compared with acyl phospholipids with a lower number of double bonds.

In summary, our data reveal wide-spread changes in the plasma lipidome—some for the first time—that occur in response to GC treatment, and highlight the similarities and differences between short- and long-term GC excess exposure in dogs. This study represents a first step toward a detailed characterisation of the influence of GCs on the lipid metabolome in dogs and other organisms. These data indicate that our dog model of tetracosactide-induced CS is a promising approach to study the effects of hypercortisolism on lipid metabolism in dogs. The relevance of our study can also be translated to human diseases, as many of the enzymes in lipid pathways that are regulated by GCs are dysregulated in chronic conditions of GC excess; as well as in e.g., metabolic syndrome and CS^54^. Our findings support the potential use of these plasma lipid classes as markers to monitor the effects of short- and long-term GC use. Finally, our results highlight that co-medication with a GC can be a significant confounding factor when analysing the plasma lipidome of patients, such as in those with auto-immune diseases (e.g., rheumatoid arthritis and lupus erythematosus)^1^. Beyond the scope of the present study, the biological role and significance of these findings will need to be evaluated in future investigations to provide deeper and novel insights of GC signalling and the impact of GCs on metabolism in health and disease.

## Methods

### Animals

Purpose-bred male and female Beagle dogs were used in both experiments. The dogs were kept in groups at the research unit of the Vetsuisse Faculty of the University of Zurich and fed a standard commercial maintenance (pellet/kibble) diet (Josera, Adult Sensitive, Kleinheubach, Germany). The experimental group treated with short-term prednisolone included 8 dogs (5 male and 3 females, 8–58 months old, 12.7 to 16.0 kg body weight). The long-term tetracosactide-treated group included 6 dogs (4 male and 2 female, 23 to 83 months old, 12.2 to 18.9 kg body weight) (see Supplementary Table S1). The study was approved by the Cantonal Veterinary Office of Zurich, Switzerland (TVB 133/2013; 276/2014) and conducted in accordance with guidelines established by the Animal Welfare Act of Switzerland.

### Short-term prednisolone treatment

Eight Beagle dogs were treated with 50 mg prednisolone (Streuli Pharma AG, Uznach, Switzerland) orally twice daily for 3 consecutive days (prednisolone group). Blood samples were collected before (control) and at 12 h after the last prednisolone treatment.

### Long-term tetracosactide treatment

Six Beagle dogs were infused subcutaneously (Alzet osmotic pump, Durect Corporation, Cupertino, CA, USA) for 25 weeks with tetracosactide (synthetic ACTH; Bachem AG, Bubendorf, Switzerland) (tetracosactide group). Every 4 weeks, new pumps were implanted subcutaneously into the dorsolateral neck under general anaesthesia to deliver increasing doses of tetracosactide. Dogs received a starting dose of 1.3–1.95 µg/kg/d tetracosactide, increasing the concentration to a final dose of 6–10 µg/kg/d. Blood samples were collected before (control) and at 25 weeks after treatment commenced.

The successful induction of hypercortisolism was tested with an ACTH stimulation test and a low-dose dexamethasone suppression test at 25 weeks after the start of treatment. For the ACTH stimulation test^22^, 5 μg/kg synthetic tetracosactide (Synacthen, Novartis Pharma Schweiz AG, Bern, Switzerland, Pharmacode: 6748610) was injected intravenously, and plasma cortisol measured at 0 and 1 h after injection. For the low-dose dexamethasone suppression (LDDS) test, 0.01 mg/kg dexamethasone (Dexadreson, MSD Animal Health GmbH, Luzern, Switzerland, ATCvet number: QH02AB02) was injected intravenously, and plasma cortisol measured at 0, 4, and 8 h after injection.

### Blood collection and processing

Blood samples were collected from the jugular vein after overnight fasting before (control) and at the end of the treatment (see above). For lipidomics analyses, blood was collected into lithium heparin tubes (Vacuette Greiner Bio-One, Frickenhausen, Germany) and centrifuged immediately after collection (1,862 *g*, 10 min, 4 °C). Obtained plasma was stored at −80 °C until analysis. For clinical chemistry analyses, serum was harvested after clot retraction at room temperature.

### Clinical chemistry

All clinical blood serum chemistry parameters were determined at the Clinical Laboratory, Vetsuisse Faculty (University of Zurich) on a Cobas Integra 800 instrument (Roche Diagnostics AG, Rotkreuz, Switzerland). The clinical chemistry parameters included triglycerides, cholesterol, alanine aminotransferase (ALAT), alkaline phosphatase (ALP), lipase and other metabolites (Fig. 1 and Supplementary Table S1). Serum cortisol concentrations were measured by a competitive immunoassay validated for dogs (DPC Immulite 1000, Siemens Schweiz AG, Zurich, Switzerland). The intra-assay coefficients of variation were 10.0% and 6.3% at cortisol levels of 2.7 and 18.9 µg/dL, respectively. The sensitivity of the assay was 0.2 µg/dL.

### Quality control samples for lipidomic analyses

For the lipidomics analyses, Process Quality Control (PQC) samples were generated for each experimental group (prednisolone and tetracosactide, respectively) by pooling equal volumes of each plasma sample within an experimental group. Aliquots (10 µL) of corresponding pooled PQC samples were then tested alongside samples of each experimental group. Blank samples, which did not contain any plasma, were also prepared and tested (see “Lipid extraction”).

### Lipid extraction

Plasma lipids were extracted using a modified version of the butanol/methanol extraction method^55^. Briefly, plasma samples were thawed on ice, and 10 µL of each sample (or PQC or control) was then transferred into a 2-mL Eppendorf tube (Eppendorf, Germany). Nothing was added to the tubes to be used as Blank samples. To prevent lipid oxidation, 1 µL of 2,6-di-tert-butyl-4-methylphenol (BHT, 10 mmol/L; Sigma-Aldrich; St Louis, MO, USA; B1378) in ethanol was added to each sample (including PQCs and Blanks). To this, 90 µL of 1-butanol:methanol (1:1, v/v) containing the internal standard lipids (Supplementary Table S8) was added. The samples were vortexed for 30 sec and sonicated in an ultrasound water bath at 20 °C for 30 min. After centrifugation (14,000 *g*, 10 min, 4 °C), 90 µL of supernatant were transferred into 1.5-mL tubes (Sarstedt Nümbrecht, Germany) and dried under a nitrogen stream at 37 °C. Samples were stored at −80 °C. Before use, samples were reconstituted in 90 µL of 1-butanol:methanol (1:1, v/v), sonicated for 10 min at room temperature, and centrifuged at 20,800 *g*, for 10 min at 4 °C. Then, 80 µL supernatant were transferred into autosampler vials with glass inserts (Agilent Technologies, Santa Clara, CA, USA) for LC-MS analysis.

### LC-MS analyses

Phospholipids, diacylglycerols, and cholesteryl esters were measured based on a published method^56^, with some modifications. Mobile phase A consisted of acetonitrile:water 4:6 (v:v) with 10 mmol/L ammonium formate (Sigma-Aldrich, 78314) and mobile phase B of acetonitrile:2-propanol 1:9 (v:v) with 10 mmol/L ammonium formate. An Agilent Zorbax RRHD Eclipse Plus C18 (2.1 × 50 mm, 1.8 µm, 95 Å) reversed-phase column maintained at 40 °C was used as the stationary phase. The gradient was composed as follows: 20% B for 2 min, 20% to 60% B from 2 to 7 min, 60% to 100% B from 7 to 9 min, back to 20% B from 9 min until the end (total runtime of 10.8 min). The flow rate was set to 0.4 mL/min and 2 µL sample was injected for analysis. The LC-MS system consisted of an Agilent 1290 infinity UHPLC pump and the Agilent 6460 triple quadrupole (QQQ) mass spectrometer. The ESI source parameters are detailed in Supplementary Table S9. All analyses were performed in dynamic MRM (multiple reaction monitoring) mode with unit resolution. Monitored transitions are listed in Supplementary Table S10.

Sphingolipids were measured using the same LC method described above. The injected sample volume was 1 µL. An Agilent QQQ 6495 was used as a mass spectrometer, with distinct ESI source settings (see Supplementary Table S9) and MRM transition list (see Supplementary Table S11).

For triacylglycerols, the stationary phase consisted of an Agilent Zorbax Eclipse XDB-C18 Silica, 3 × 150 mm, 1.8 μm, 80 Å column maintained at 25 °C. LC separation was performed isocratically for 25 min at a flow rate of 128 μL/min with chloroform:methanol 1:1 (v:v) containing 2 mmol/L ammonium acetate (Sigma-Aldrich, 17843) as the mobile phase. The LC-MS system consisted of an Agilent 1100 HPLC pump and a Sciex 4000 QTrap mass spectrometer (SCIEX, Framingham, MA, USA) operated in single-ion monitoring (SIM) mode at unit resolution to measure TG precursor ions (see Supplementary Table S12). The ESI source settings are described in Supplementary Table S9. The injected sample volume was 10 µL.

Sphingosine-1-phosphate (S1P) analysis was performed according to the method described by Narayanaswamy *et al*.^35^. To 50 µL of reconstituted lipid extract, 50 µL of ^13^C_2_D_2_–S1P d18:1 internal standard (20 ng/mL; Toronto Research Chemicals, Toronto, Canada) in 1-butanol:methanol (1:1, v/v) was added, and the sample was derivatized by the addition of 20 µL of TMS-diazomethane (2 mol/L in hexanes; Acros Organics, Thermo Fisher Scientific, New Jersey, USA) for 20 min at 25 °C and 700 rpm (Thermomixer, Eppendorf, Germany). The reaction was stopped by the addition of 1 µL of 100% acetic acid. Samples were centrifuged at 20,800 *g* for 10 min at 7 °C, and supernatants transferred into autosampler vials for subsequent LC-MS analysis. The mobile phase A consisted of 1:1 (v/v) acetonitrile:25 mmol/L ammonium formate solution (in water, adjusted to pH 4.6 with formic acid), and the mobile phase B of 95:5 (v:v) acetonitrile: 25 mmol/L ammonium formate solution. Analytes were eluted with the following gradient: 99.9% B from 0 to 5 min; 40% B 5 to 5.5 min; 10% B 5.5 to 6.6 min and 99.9% B 6.6 to 9.6 min (total run time 9.6 min). The flow rate was 0.4 mL/min. Samples (5 µL) were injected onto a Waters (Milford, USA) ACQUITY UPLC BEH HILIC (2.1 × 100 mm, 1.7 µm, 130 Å) analytical column maintained at 60 °C. The MS system consisted of an Agilent QQQ 6490. The ESI source parameters are indicated in Supplementary Table S9, and the monitored MRM transition list in Supplementary Table S13. Data acquisition was performed using Agilent MassHunter software (version B.06).

### MS data processing

The TG raw data was processed with Analyst (Version 1.6.2, SCIEX), and all other data from Agilent instruments with MassHunter QQQ Quantitative software (version B.08). The retention time and, if available, qualifier transitions were used to assign peaks to corresponding lipids. For sphingolipids, transitions of precursor ions with water loss were used as qualifiers. For S1P, the m/z 60 transitions were used as quantifiers and the interference from the S1P d18:1 M + 2 isotope was subtracted from S1P d18:0^57^. For plasmalogen PE (PE-P), transitions with the fatty acid as product were used as quantifiers, and those with the head group as qualifiers. Plasmalogen PCs (PC-P), ether PCs (PC-O) and odd-chain fatty acid PCs were distinguished based on retention time^56^.

Normalised peak areas were calculated by dividing the peak areas of the analyte with the corresponding internal standard (ISTD; see Supplementary Table S14). Relative abundance was obtained by multiplying the normalised peak areas with the molar concentration of the corresponding ISTD (see Supplementary Table S8). Lipid species with a median peak area in the PQC samples below 250 or less than 5 times of the Blank samples were excluded. Additionally, the coefficient of variation (CV) of the normalised peak area was calculated for each lipid species in the PQC samples of each experimental group. Species with a CV higher than 25% in any of the two groups were excluded from subsequent evaluation, except of Cer d18:1/18:0 and Hex1Cer d18:1/24:1, which had CVs of 27.8% and 30.1%, respectively, in the tetracosactide group, and 7.3% and 10.9% in the prednisolone group. These species were kept in the dataset to allow comparison between the two groups. The final filtered dataset included 262 lipid species quantified in all samples of both experimental groups (see Supplementary Table S2). The median %CVs of lipids species were 5.3% and 4.5% in the phospholipid/CE/DG, 12.1% and 10.9% in the sphingolipid, 8.5% and 13.1% in the S1P, and 18.1% and 18.4% in the TG panel analyses of the prednisolone and tetracosactide group, respectively. All calculations were performed using R^58^ (see Data Availability).

### Statistics and visualization

Statistical significance of changes in measured clinical chemistry parameters, body weights and lipid abundances between dogs before and after treatment were determined by paired, two-tailed *t*-tests from log2-transformed values. FDR (false discovery rate)-adjusted *P* values were calculated using the Benjamini–Hochberg procedure. All calculations and figures were generated using R scripts using R^58^ and following described R packages (see Data Availability). Heatmaps of the lipid abundances from samples before and after treatments (Fig. 2a,b) were generated using the heatmap.2 function of the gplots^59^ R package with Pearson’s distance and Ward clustering algorithms. The scales indicate autoscaled log2-fold changes. PCA plots were generated with the R packages FactoMineR^60,61^ and factoextra^62^ from scaled, centred log2-transformed lipid abundances (Fig. 2c,d) and log2-folds changes (Fig. 2e,f). The heatmaps of fold changes and FDR-adjusted *P* values (Figs 3 to 6) were generated using ComplexHeatmap^63–65^. Other plots were generated using ggplot2^66^. Figures were scaled and further annotated in Adobe Illustrator CC (Adobe Systems, San Jose, CA).

## Supplementary information

Supplementary Tables and Figures S1, S4, S8-S14

Supplementary Tables S2, S3, S5-S7

## Data Availability

The lipidomics dataset including the mass spectrometry raw data has been deposited to the Metabolomics Workbench^67^ (Project ID: PR000761) and is accessible via the DOI: 10.21228/M89Q32^68^. All R scripts used to generate the data and figures in this manuscript are available from Github (https://github.com/SLINGhub/Manuscript_Sieber-Ruckstuhl_Burla_2019)^69^. The version of the Github repository at the time of writing this manuscript has been archived in Zenodo (DOI: 10.5281/zenodo.2581113)^70^.
