# Supplementary material for: Changes in the Canine Plasma Lipidome after Short- and Long-Term Excess Glucocorticoid Exposure
Source: Sci Rep. 2019 Apr 12;9:6015. doi: 10.1038/s41598-019-42190-1 (PMC6461633; doi:10.1038/s41598-019-42190-1)
Supplement: Supplementary file 1 — Supplementary Tables and Figures S1, S4, S8-S14 [file 41598_2019_42190_MOESM1_ESM.pdf]

Supplementary Information

Changes in the Canine Plasma Lipidome after Short- and Long-Term Excess Glucocorticoid Exposure

Nadja S. Sieber-Ruckstuhl[1#*], Bo Burla[2#], Susanne Spoerel[1], Florence Schmid[1], Claudio Venzin[4], Amaury Cazenave-Gassiot[3], Anne K. Bendt[2], Federico Torta[3*], Markus R. Wenk[2,3], Felicitas S. Boretti[1*]

[1] Clinic for Small Animal Internal Medicine, Vetsuisse Faculty, University of Zurich Switzerland

[2] Singapore Lipidomics Incubator (SLING), Life Sciences Institute, National University of Singapore, Singapore

[3] Department of Biochemistry, YLL School of Medicine, National University of Singapore, Singapore

[4] Clinic for Small Animal Surgery, Vetsuisse Faculty, University of Zurich, Switzerland

[#] N. Sieber-Ruckstuhl and B. Burla contributed equally to this work

[*] To whom correspondence should be addressed

Supplementary Table S1: Biographic and clinical parameters of dogs before and after treatments. Prednisolone treatment is indicated as PRED and tetracosactide as TET. The laboratory's device-specific reference intervals were: lipase 24-108 U/L, trigylcerides 0.4-1.5 mmol/L, cholesterol 3.5-8.6 mmol/L, alanine aminotransferase 20-93 U/L, alkaline phosphatase 20-98 U/L, creatine kinase 51-191 U/L, sodium 145-152 mmol/L, potassium 4.3-5.3 mmol/L, chloride 107-118 mmol/L, calcium 2.4-2.8 mmol/L, phosphate 1-1.6 mmol/L, bilirubin total 0-3.5 µmol/L, glucose 4.1-5.9 mmol/L, fructosamine <340 µmol/L, urea 3.8-9.4 mmol/L, creatinine 50-119 µmol/L, protein 56-71 g/L, albumin 29-37 g/L. No reference interval was defined for cortisol. ND indicates that parameter was not determined, NQ that parameter was not quantifiable in this sample.

Animal ID	Treatment	Time	Sex	Age (months)	Weight (kg)	Cortisol (µg/dL)	Lipase (U/L)	Triglycerides (mmol/L)	Cholesterol (mmol/L)	Alanine aminotransferase (U/L)	Alkaline phosphatase (U/L)	ACTH/LDDS Test Results	Polyuria	Polydipsia	Polyphagia	Muscle Wasting	Central Obesity	Creatine kinase (U/L)	Sodium (mmol/L)	Potassium (mmol/L)	Chloride (mmol/L)	Calcium (mmol/L)	Phosphate (mmol/L)	Bilirubin Total (µmol/L)	Glucose (mmol/L)	Fructosamin (µmol/L)	Urea (mmol/L)	Creatinine (µmol/L)	Protein (g/L)	Albumin (g/L)	Globulin (g/L)
T1	TET	before	M	83	14.7	2.5	23	0.4	4.5	37	47	ND	No	No	No	No	No	167	147	4.1	112	2.53	1.2	2.5	5.4	268	3.5	55	59	35	24
T1	TET	after	M	89	12.6	7.0	57	1.3	6.6	127	206	+/+	Yes	Yes	No	No	No	248	146	4.3	107	2.34	1.71	2.5	5.3	194	2.1	19	55	25	30
T2	TET	before	F	72	14.0	2.5	42	0.5	4.9	45	62	ND	No	No	No	No	No	128	146	4	109	2.71	1.26	2.5	5.2	288	3.1	48	63	37	26
T2	TET	after	F	78	11.8	46.3	194	0.6	4.5	326	1004	+/+	Yes	Yes	No	Yes	Yes	151	145	4.1	100	2.49	1.27	2.5	6.8	233	1.2	NQ	60	36	24
T3	TET	before	F	71	15.4	1.9	40	0.4	6.1	44	57	ND	No	No	No	No	No	107	146	3.9	111	2.57	1.22	2.5	5.5	287	2.9	62	57	36	21
T3	TET	after	F	77	12.2	10.1	1866	1	8.1	672	665	+/+	Yes	Yes	No	Yes	Yes	154	140	4.7	96	2.34	1.28	2.5	6.9	205	1.5	16	56	33	23
T4	TET	before	M	23	16.9	2.6	22	0.2	4.7	44	41	ND	No	No	No	No	No	691	150	3.7	110	2.77	1.39	2.5	6.0	304	3.7	75	61	37	25
T4	TET	after	M	29	14.1	8.6	397	0.3	2.7	406	182	+/+	Yes	Yes	No	Yes	No	201	146	3.9	102	2.62	1.12	2.5	5.1	238	2.3	27	59	36	24
T5	TET	before	M	23	19.8	5.4	48	0.2	5.5	73	47	ND	No	No	No	No	No	599	151	3.4	112	2.76	1.43	2.5	7.1	326	4.1	81	65	40	25
T5	TET	after	M	29	16.5	9.5	141	0.4	5	154	161	+/+	Yes	Yes	No	Yes	No	92	146	3.9	103	2.58	1.27	2.5	5.6	248	3.0	31	61	39	22
T6	TET	before	M	88	16.3	11.9	28	0.2	5.6	64	68	ND	No	No	No	No	No	565	147	3.7	109	2.61	1.22	2.5	6.3	272	4.1	56	62	34	27
T6	TET	after	M	94	11.9	16.1	91	3.3	9.5	140	685	+/+	No	No	No	No	No	135	151	3.5	111	2.12	2.62	2.5	5.9	191	6.0	71	47	15	33
P1	PRED	before	F	58	12.7	1.7	41	0.8	5.5	31	42	ND	No	No	No	No	No	100	144	3.6	105	2.60	1.09	2.5	5.2	243	4.1	51	59	38	21
P1	PRED	after	F	58	ND	0.3	85	1.2	5.8	70	114	ND	Yes	Yes	Yes	No	No	48	148	3.8	98	2.59	1.22	2.5	4.0	273	4.2	47	63	41	22
P2	PRED	before	F	43	13.5	1.8	77	1.4	4.7	35	109	ND	No	No	No	No	No	144	147	3.7	109	2.59	1.19	2.5	5.8	281	4.7	48	62	38	24
P2	PRED	after	F	43	ND	0.3	104	0.5	3.4	49	157	ND	Yes	Yes	Yes	No	No	140	150	3.2	103	2.37	1.10	2.5	5.3	284	4.5	41	64	41	23
P3	PRED	before	F	56	15.0	1.3	52	0.7	6.3	35	58	ND	No	No	No	No	No	221	149	3.6	112	2.55	1.36	2.5	5.5	264	4.1	69	59	38	21
P3	PRED	after	F	56	ND	0.3	78	0.7	4.5	51	139	ND	Yes	Yes	Yes	No	No	89	147	4.1	100	2.58	1.42	2.5	5.3	278	4.4	48	63	42	21
P4	PRED	before	M	43	15.0	1.5	54	0.6	5.7	63	40	ND	No	No	No	No	No	140	148	3.7	108	2.60	1.33	2.5	6.3	305	5.7	69	61	39	22
P4	PRED	after	M	43	ND	2.3	107	0.3	4.1	151	113	ND	Yes	Yes	Yes	No	No	77	151	3.7	99	2.35	1.52	2.5	5.3	263	5.8	50	55	33	22
P5	PRED	before	M	8	14.5	1.6	40	0.3	5.0	35	74	ND	No	No	No	No	No	172	147	3.9	110	2.81	2.23	2.5	5.4	244	5.0	73	55	39	16
P5	PRED	after	M	8	ND	0.5	32	0.3	5.7	178	160	ND	Yes	Yes	Yes	No	No	141	152	4.2	93	2.55	2.60	5.1	3.4	239	4.4	49	58	24	34
P6	PRED	before	M	7	14.0	1.1	29	0.5	6.0	29	93	ND	No	No	No	No	No	166	146	3.7	109	2.80	2.01	2.5	5.5	241	5.2	76	55	35	19
P6	PRED	after	M	7	ND	1.4	123	0.2	4.0	57	84	ND	Yes	Yes	Yes	No	No	118	154	3.9	93	2.46	3.48	9.1	2.7	223	6.0	40	55	23	31
P7	PRED	before	M	7	16.0	1.1	56	0.3	5.7	47	71	ND	No	No	No	No	No	190	147	3.8	111	2.65	1.97	2.5	5.5	282	5.9	77	52	38	15
P7	PRED	after	M	7	ND	0.4	106	0.2	5.7	111	94	ND	Yes	Yes	Yes	No	No	75	150	3.8	99	2.87	1.46	2.5	3.7	280	5.6	58	60	41	19
P8	PRED	before	M	8	14.0	1.6	25	0.4	6.9	32	72	ND	No	No	No	No	No	108	143	3.7	108	2.70	1.78	2.5	5.6	260	5.9	76	55	36	20
P8	PRED	after	M	8	ND	0.5	56	0.5	6.9	70	101	ND	Yes	Yes	Yes	No	No	89	150	3.8	95	2.84	1.39	2.5	4.1	294	5.1	55	64	41	23

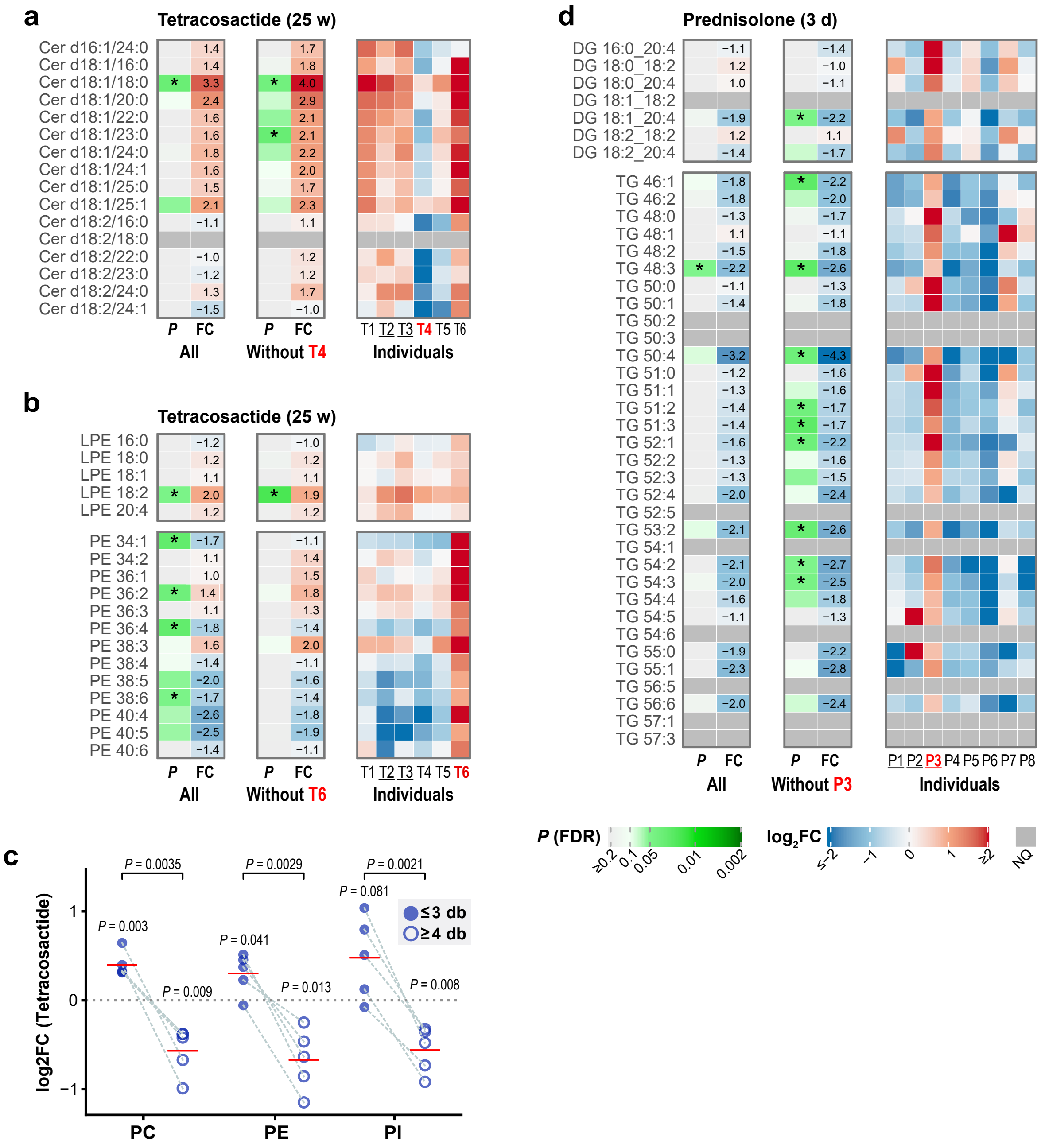

Supplementary Figure S4: Comparison of fold changes and FDR-adjusted *P* values for all individuals and when specific dogs were excluded from the analysis. (a) Effect of excluding dog T4 in the tetracosactide group on the test statistics of the ceramides. **(b)** Effect of excluding dog T6 in the etracosactide group on the test statistics of lyso-phosphatidylethanolamines (LPE) and PE. **(c)** Changes in total levels of PC, PE and PI lipid species with either 0 to 3 (≤ 3, filled circles) or 4 to 8 (≥ 4, open circles) fatty acid double bonds after long-term tetracosactide treatment when dog T6 is excluded from the analysis. See Figure 5b in the main text for details. **d)** Effect of excluding dog P3 in the prednisolone group on the test statistics of diacylglycerols (DG) and triacylglycerols (TG). FDR-adjusted P values, obtained from paired two-tailed t-tests comparing log-transformed concentrations before and after treatment (*P*; n.s. $P > 0.05$, *$P \leq 0.05$, **$P \leq 0.01$), and the fold changes (FC) after vs. before treatment are indicated for the full dataset (All) and for datasets excluding the indicated dog (Without). The log2FC of the individual dogs (P1-P8 for prednisolone, T1-T6 for tetracosactide) are also indicated (Individuals). The color scale in FC corresponds to log2-fold changes (log2FC), and the values to FC. |FC| > 4 are capped at the color scale with the maximum red and blue color. Female dogs are underlined. Dark grey rows (NQ) indicate lipid species did not meet the quality control criteria. Underlying data are shown in Supplementary Tables S4-S6.

Supplementary Table S8: Internal standards and their concentrations in the BuMe-ISTD extraction mix.

Lipid standards were prepared as 1 mg/mL stock solutions in chloroform:methanol (2:1, v/v) and then mixed in 1-butanol:methanol (1:1, v/v).

Short Name	Full Name/Description	Transition Name	Concentration [ng/mL]	Supplier Cat. No.
**TAG 16:0_16:0_16:0 d5**	Glyceryl-d5 Trihexadecanoate	d5 TAG 48:0 (IS)	100	CDN Isotopes D-5815
**DG 12:0_12:0**	1,2-dilauroyl-sn-glycerol	DG 24:0 (12:0) (IS)	100	Avanti 800812P
**PC 14:0/14:0**	1,2-dimyristoyl-sn-glycero-3-phosphocholine	PC 28:0 (IS)	100	Avanti 850345P
**PI 12:0/13:0**	1-dodecanoyl-2-tridecanoyl-sn-glycero-3-phospho-(1'-myo-inositol) (ammonium salt)	PI 25:0 (IS)	50	Avanti LM-1500
**PE 14:0/14:0**	1,2-dimyristoyl-sn-glycero-3-phosphoethanolamine	PE 28:0 (IS)	50	Avanti 850745P
**SM d18:1/C12:0**	N-lauroyl-D-erythro-sphingosylphosphorylcholine	SM 30:1 (IS)	50	Avanti 860583P
**Cer d18:1/C17:0**	N-heptadecanoyl-D-erythro-sphingosine	Cer d18:1/17:0 (IS)	50	Avanti 860517P
**LPC 20:0**	1-arachidoyl-2-hydroxy-sn-glycero-3-phosphocholine	LPC 20:0 (IS)	50	Avanti 855777P
**LPE 14:0**	1-myristoyl-2-hydroxy-sn-glycero-3-phosphoethanolamine	LPE 14:0 (IS)	50	Avanti 856735P
**DHC 16:0 d3**	Lactosylceramide 16:0 d3	DHCer d18:1/16:0 d3 (IS)	50	Matreya 1534
**MHC d18:1/C16:0 d3**	N-omega-CD3-Hexadecanoyl-glucopsychosine	MHC d18:1/16:0 d3 (IS)	50	Matreya 1533
**PS 14:0/14:0**	1,2-dimyristoyl-sn-glycero-3-phospho-L-serine (sodium salt)	PS 28:0 (IS)	50	Avanti 840033P

Supplementary Table S9: ESI source parameter settings for the Agilent and Sciex mass spectrometers

	**Instrument Method**			
**Source Parameter**	Agilent QQQ6460 Phospholipids, DG, CE	Agilent QQQ6495 Sphingolipids		Agilent QQQ6490 S1P
Polarity	Positive	Positive	Negative	Positive
Gas Temperature (°C)	300	200	200	200
Gas Flow (L/min)	5	12	12	12
Nebuliser (psi)	45	25	25	25
Sheath Gas Temperature (°C)	250	250	250	400
Sheath Gas Flow (L/min)	11	12	12	12
Capillary Voltage (V)	3500	3500	3000	3500
Noozle	500	500	1500	500
iFunnel High Pressure RF	N/A	80	150	200
iFunnel Low Pressure RF	N/A	40	60	110

	**Instrument Method**
**Source Parameter**	Sciex API4000 TG
Polarity	Positive
Electrospray voltage (kV)	5
Source temperature (°C)	250
Drying gas	Nitrogen
Gas 1 flow	40
Gas 2 flow	30
Curtain gas flow	10

Supplementary Table S10: Transition list for the phospholipids, diacylglycerols and cholesteryl esters method.

Compound Name	Precursor m/z	Product m/z	Ret. Time (min)	Delta RT (min)	Collision Energy	Cell Accelerator Voltage	Polarity
CE 14:0	614.6	369.3	8.21	0.70	10	5	Positive
CE 15:0	628.6	369.3	8.40	0.70	10	5	Positive
CE 16:0	642.6	369.3	8.50	0.70	10	5	Positive
CE 16:1	640.6	369.3	8.20	0.70	10	5	Positive
CE 16:2	638.6	369.3	8.10	0.70	10	5	Positive
CE 17:0	656.6	369.3	8.50	0.70	10	5	Positive
CE 17:1	654.6	369.3	8.40	0.70	10	5	Positive
CE 18:0	670.7	369.3	8.38	0.70	10	5	Positive
CE 18:1	668.6	369.3	8.30	0.50	10	5	Positive
CE 18:2	666.6	369.3	8.20	0.70	10	5	Positive
CE 18:3	664.6	369.3	8.10	0.70	10	5	Positive
CE 20:1	696.7	369.3	8.60	0.70	10	5	Positive
CE 20:2	694.7	369.3	8.36	0.70	10	5	Positive
CE 20:3	692.6	369.3	8.15	0.70	10	5	Positive
CE 20:4	690.6	369.3	8.20	0.70	10	5	Positive
CE 20:5	688.6	369.3	8.00	0.70	10	5	Positive
CE 22:0	726.7	369.3	8.90	1.00	10	5	Positive
CE 22:1	724.7	369.3	8.61	1.00	10	5	Positive
CE 22:4	718.7	369.3	8.25	0.70	10	5	Positive
CE 22:5	716.6	369.3	8.12	0.70	10	5	Positive
CE 22:6	714.6	369.3	8.00	0.70	10	5	Positive
CE 24:0	754.7	369.3	9.20	0.70	10	5	Positive
CE 24:1	752.7	369.3	8.90	0.70	10	5	Positive
CE 24:4	746.7	369.3	8.40	0.70	10	5	Positive
CE 24:5	744.7	369.3	8.40	0.70	10	5	Positive
CE 24:6	742.7	369.3	8.30	0.70	10	5	Positive
DG 24:0 -(12:0) (IS)	474.7	257.1	4.92	0.70	10	7	Positive
DG 30:0 -(14:0)	558.5	313.3	6.20	0.70	21	5	Positive
DG 30:0 -(16:0)	558.5	285.2	6.20	0.70	21	5	Positive
DG 30:1 -(14:0)	556.5	311.3	5.90	0.70	21	5	Positive
DG 30:1 -(16:1)	556.5	285.2	5.90	0.70	21	5	Positive
DG 32:0 -(14:0)	586.5	341.3	6.50	0.80	21	5	Positive
DG 32:0 -(16:0)	586.5	313.2	6.50	0.80	21	5	Positive
DG 32:0 -(18:0)	586.5	285.2	6.50	0.80	21	5	Positive
DG 32:1 -(14:0)	584.5	339.3	6.20	0.80	21	5	Positive
DG 32:1 -(16:0)	584.5	311.2	6.20	0.80	21	5	Positive
DG 32:1 -(16:1)	584.5	313.2	6.30	0.80	21	5	Positive
DG 32:1 -(18:1)	584.5	285.2	6.20	0.80	21	5	Positive
DG 32:2 -(14:0)	582.5	337.3	5.85	0.70	21	5	Positive
DG 32:2 -(16:1)	582.5	311.2	5.98	0.70	21	5	Positive
DG 32:2 -(18:2)	582.5	285.2	5.95	0.70	21	5	Positive
DG 34:0 -(16:0)	614.6	341.3	6.90	0.80	21	5	Positive
DG 34:0 -(18:0)	614.6	313.3	6.70	0.80	21	5	Positive
DG 34:1 -(16:0)	612.6	339.3	6.55	0.70	21	5	Positive
DG 34:1 -(18:0)	612.6	311.3	6.50	0.70	21	5	Positive
DG 34:1 -(18:1)	612.6	313.3	6.40	1.00	21	5	Positive
DG 34:1 -(20:0)	612.6	283.3	6.00	0.70	21	5	Positive
DG 34:2 -(16:0)	610.5	337.2	6.28	0.80	21	5	Positive
DG 34:2 -(16:1)	610.5	339.2	6.21	0.80	21	5	Positive
DG 34:2 -(18:1)	610.5	311.2	6.35	0.80	21	5	Positive
DG 34:2 -(18:2)	610.5	313.2	6.35	0.80	21	5	Positive
DG 36:0 -(16:0)	642.7	369.3	7.10	0.70	21	5	Positive
DG 36:0 -(18:0)	642.6	341.3	7.10	0.70	21	5	Positive
DG 36:0 -(20:0)	642.6	313.3	7.10	0.70	21	5	Positive
DG 36:1 -(18:0)	640.6	339.3	6.70	0.80	21	5	Positive
DG 36:1 -(18:1)	640.6	341.3	6.66	0.80	21	5	Positive
DG 36:2 -(18:0)	638.6	337.3	6.50	0.80	21	5	Positive
DG 36:2 -(18:1)	638.6	339.3	6.50	0.80	21	5	Positive
DG 36:2 -(18:2)	638.6	341.3	6.50	0.80	21	5	Positive
DG 36:3 -(18:1)	636.6	337.3	6.30	0.70	21	5	Positive
DG 36:3 -(18:2)	636.6	339.3	6.30	0.70	21	5	Positive
DG 36:3 -(20:3)	636.6	313.3	6.40	0.70	21	5	Positive
DG 36:4 -(18:2)	634.5	337.2	6.15	0.70	21	5	Positive
DG 36:4 -(18:3)	634.5	339.2	6.16	0.70	21	5	Positive
DG 36:4 -(20:4)	634.5	313.2	6.23	0.70	21	5	Positive
DG 38:1 -(18:1)	668.7	369.3	6.73	0.80	21	5	Positive
DG 38:4 -(20:3)	662.6	339.3	6.50	0.70	21	5	Positive
DG 38:4 -(20:4)	662.6	341.3	6.50	0.70	21	5	Positive
DG 38:5 -(18:1)	660.6	361.3	6.17	0.80	21	5	Positive
DG 38:5 -(20:3)	660.6	337.3	6.25	0.80	21	5	Positive
DG 38:5 -(20:4)	660.6	339.3	6.38	0.80	21	5	Positive
DG 38:5 -(22:5)	660.6	313.3	6.35	0.80	21	5	Positive
DG 38:6 -(20:4)	658.5	337.2	6.13	0.80	21	5	Positive
DG 38:6 -(22:6)	658.5	313.2	6.23	0.80	21	5	Positive
LPC 14:0	468.3	184.1	2.18	0.80	21	5	Positive
LPC 15:0	482.3	184.1	2.55	0.80	21	5	Positive
LPC 16:0	496.3	184.1	2.85	0.80	21	5	Positive
LPC 16:1	494.3	184.1	2.50	1.00	21	5	Positive
LPC 17:0	510.35	184.1	3.17	0.80	21	5	Positive
LPC 17:1	508.4	184.1	2.75	1.00	21	5	Positive
LPC 18:0	524.4	184.1	3.46	0.80	21	5	Positive
LPC 18:1	522.4	184.1	3.00	0.80	21	5	Positive
LPC 18:2	520.3	184.1	2.63	1.00	21	5	Positive

Compound Name	Precursor m/z	Product m/z	Ret. Time (min)	Delta RT (min)	Collision Energy	Cell Accelerator Voltage	Polarity
LPC 18:3	518.3	184.1	2.30	1.00	21	5	Positive
LPC 19:0	538.4	184.1	3.63	0.80	21	5	Positive
LPC 20:0 (IS)	552.4	184.1	3.87	0.80	21	5	Positive
LPC 20:1	550.4	184.1	3.45	0.80	21	5	Positive
LPC 20:2	548.4	184.1	3.00	1.00	21	5	Positive
LPC 20:3	546.4	184.1	2.75	1.10	21	5	Positive
LPC 20:4	544.3	184.1	2.65	0.80	21	5	Positive
LPC 20:5	542.3	184.1	2.25	0.80	21	5	Positive
LPC 22:0	580.4	184.1	4.30	0.80	21	5	Positive
LPC 22:1	578.4	184.1	3.80	0.80	21	5	Positive
LPC 22:5	570.4	184.1	2.83	0.80	21	5	Positive
LPC 22:6	568.3	184.1	2.55	0.80	21	5	Positive
LPC 24:0	608.5	184.1	4.80	0.80	21	5	Positive
LPC 26:0	636.5	184.1	5.15	0.80	21	5	Positive
LPC O-16:0	482.4	104.1	2.95	1.00	21	5	Positive
LPC O-18:0	510.4	104.1	3.40	1.00	21	5	Positive
LPC O-18:1	508.4	104.1	3.17	1.30	21	5	Positive
LPC O-20:0	538.4	104.1	3.62	1.00	21	5	Positive
LPC O-20:1	536.4	104.1	3.30	1.20	21	5	Positive
LPC O-22:0	566.5	104.1	4.60	0.80	21	5	Positive
LPC O-22:1	564.4	104.1	4.25	0.78	21	5	Positive
LPC O-24:0	594.5	104.1	5.10	1.00	21	5	Positive
LPC O-24:1	592.5	104.1	4.75	0.70	21	5	Positive
LPC O-24:2	590.5	104.1	4.00	0.88	21	5	Positive
LPE 14:0 (IS)	426.3	285.2	2.20	0.70	17	5	Positive
LPE 16:0	454.3	313.3	3.00	0.70	17	5	Positive
LPE 18:0	482.3	341.3	3.25	1.00	17	5	Positive
LPE 18:1	480.3	339.3	3.07	0.80	17	5	Positive
LPE 18:2	478.3	337.3	2.55	0.80	17	5	Positive
LPE 20:4	502.3	361.3	2.60	0.80	17	5	Positive
LPE 22:6	526.3	385.3	2.52	0.80	17	5	Positive
LPI 18:0	618.3	341.3	2.70	0.80	17	5	Positive
LPI 18:1	616.3	339.3	2.40	0.80	17	5	Positive
LPI 18:2	614.3	337.3	2.00	0.98	17	5	Positive
LPI 20:4	638.3	361.3	2.03	1.00	17	5	Positive
PC 28:0 (IS)	678.5	184.1	4.93	0.70	21	5	Positive
PC 29:0	692.5	184.1	5.10	1.20	21	5	Positive
PC 30:0	706.5	184.1	5.35	0.70	21	5	Positive
PC 31:0 | PC O-32:0	720.6	184.1	5.65	1.59	21	5	Positive
PC 31:1 | PC O-32:1 | PC P-32:0	718.5	184.1	5.65	1.62	21	5	Positive
PC 32:0	734.6	184.1	5.67	0.80	21	5	Positive
PC 32:1	732.6	184.1	5.51	0.80	21	5	Positive
PC 32:2	730.5	184.1	5.40	1.00	21	5	Positive
PC 32:3	728.5	184.1	5.13	1.00	21	5	Positive
PC 33:0	748.6	184.1	5.90	1.20	21	5	Positive
PC 33:1 | PC O-34:1	746.6	184.1	5.90	1.55	21	5	Positive
PC 33:2 | PC O-34:2 | PC P-34:1	744.6	184.1	5.40	1.50	21	5	Positive
PC 33:3 | PC O-34:3 | PC P-34:2	742.5	184.1	5.50	1.50	21	5	Positive
PC 34:0	762.6	184.1	5.85	0.80	21	5	Positive
PC 34:1	760.6	184.1	5.73	0.80	21	5	Positive
PC 34:2	758.6	184.1	5.51	0.80	21	5	Positive
PC 34:3	756.6	184.1	5.21	0.80	21	5	Positive
PC 34:4	754.5	184.1	4.95	1.20	21	5	Positive
PC 34:5	752.5	184.1	4.65	1.20	21	5	Positive
PC 35:0 | PC O-36:0	776.6	184.1	5.40	1.60	21	5	Positive
PC 35:1 | PC O-36:1	774.6	184.1	5.30	1.60	21	5	Positive
PC 35:2 | PC O-36:2	772.6	184.1	5.80	1.30	21	5	Positive
PC 35:3 | PC O-36:3 | PC P-36:2	770.6	184.1	5.65	1.70	21	5	Positive
PC 35:4 | PC O-36:4	768.6	184.1	5.57	1.18	21	5	Positive
PC 35:5	766.5	184.1	5.65	0.70	21	5	Positive
PC 36:0 | PC P-38:6	790.6	184.1	5.91	1.30	21	5	Positive
PC 36:1	788.6	184.1	6.15	1.20	21	5	Positive
PC 36:2	786.6	184.1	5.87	1.00	21	5	Positive
PC 36:3	784.6	184.1	5.54	1.00	21	5	Positive
PC 36:5	780.6	184.1	5.20	0.80	21	5	Positive
PC 36:6	778.5	184.1	4.95	1.20	21	5	Positive
PC 37:4 | PC O-38:4	796.6	184.1	5.80	1.01	21	5	Positive
PC 37:5 | PC O-38:5 | PC P-38:4	794.6	184.1	5.63	1.70	21	5	Positive
PC 37:6 | PC P-38:5	792.6	184.1	5.60	1.61	21	5	Positive
PC 38:2	814.6	184.1	6.25	1.00	21	5	Positive
PC 38:3	812.6	184.1	5.95	1.00	21	5	Positive
PC 38:4	810.6	184.1	5.74	1.00	21	5	Positive
PC 38:5	808.6	184.1	5.46	1.00	21	5	Positive
PC 38:7	804.6	184.1	4.90	1.00	21	5	Positive
PC 39:5 | PC O-40:5	822.6	184.1	5.94	1.55	21	5	Positive
PC 39:6 | PC O-40:6	820.6	184.1	5.80	1.57	21	5	Positive
PC 39:7 | PC O-40:7 | PC P-40:6	818.6	184.1	5.61	1.69	21	5	Positive
PC 40:4	838.6	184.1	5.96	1.00	21	5	Positive
PC 40:5	836.6	184.1	5.79	0.70	21	5	Positive
PC 40:6	834.6	184.1	5.72	0.80	21	5	Positive
PC 40:7	832.6	184.1	5.37	1.00	21	5	Positive
PC 40:8	830.6	184.1	5.15	0.70	21	5	Positive
PC 36:4	782.6	184.1	5.59	0.80	21	5	Positive
PC 38:6	806.6	184.1	5.23	1.14	21	5	Positive
PC O-32:2 | PC P-32:1	716.6	184.1	5.39	1.34	21	5	Positive
PC O-34:4 | PC P-34:3	740.6	184.1	5.51	1.09	21	5	Positive

Compound Name	Precursor m/z	Product m/z	Ret. Time (min)	Delta RT (min)	Collision Energy	Cell Accelerator Voltage	Polarity
PC O-36:5 | PC P-36:4	766.6	184.1	5.54	1.00	21	5	Positive
PC P-30:0	690.4	184.1	5.16	0.70	21	5	Positive
PC P-36:5	764.6	184.1	5.40	0.70	21	5	Positive
PE 28:0 (IS)	636.5	495.5	5.06	0.70	17	5	Positive
PE 32:0	692.5	551.5	5.67	0.70	17	5	Positive
PE 32:1	690.5	549.5	5.48	0.70	17	5	Positive
PE 34:0	720.6	579.5	6.19	0.70	17	5	Positive
PE 34:1	718.5	577.5	5.80	0.80	17	5	Positive
PE 34:2	716.5	575.5	5.55	0.70	17	5	Positive
PE 34:3	714.5	573.5	5.29	0.70	17	5	Positive
PE 35:1	732.6	591.5	6.20	1.20	17	5	Positive
PE 35:2 | PE O-36:2 | PE P-18:0/18:1	730.5	589.5	6.10	1.20	17	5	Positive
PE 36:0	748.6	607.6	6.22	0.70	17	5	Positive
PE 36:1	746.6	605.6	6.09	0.70	17	5	Positive
PE 36:2	744.6	603.5	5.90	0.70	17	5	Positive
PE 36:3	742.5	601.5	5.59	1.00	17	5	Positive
PE 36:4	740.5	599.5	5.52	0.70	17	5	Positive
PE 36:5	738.5	597.5	5.33	0.70	17	5	Positive
PE 38:3	770.6	629.6	5.94	0.70	17	5	Positive
PE 38:4	768.6	627.5	5.86	0.70	17	5	Positive
PE 38:5	766.5	625.5	5.60	0.70	17	5	Positive
PE 38:6	764.5	623.5	5.47	0.70	17	5	Positive
PE 40:4	796.6	655.6	6.05	0.70	17	5	Positive
PE 40:5	794.6	653.6	5.91	0.70	17	5	Positive
PE 40:6	792.6	651.5	5.87	0.70	17	5	Positive
PE 40:7	790.5	649.5	5.59	0.70	17	5	Positive
PE O-18:0/22:5 | PE P-20:0/20:4	780.6	639.6	6.14	1.16	17	5	Positive
PE O-18:1/18:2 | PE P-18:0/18:2	728.6	587.5	5.96	0.97	17	5	Positive
PE O-18:1/20:3	754.6	613.6	6.05	1.00	17	5	Positive
PE O-18:2/18:2	726.5	585.5	5.80	0.70	17	5	Positive
PE O-18:2/20:3 | PE P-18:0/20:4	752.6	611.5	6.08	1.04	17	5	Positive
PE O-18:2/22:5 | PE P-18:0/22:6	776.6	635.5	6.06	1.02	17	5	Positive
PE O-34:1	704.6	563.5	6.10	0.70	17	5	Positive
PE O-34:2 | PE P-16:0/18:1	702.5	561.5	5.94	1.07	17	5	Positive
PE O-36:5 | PE P-16:0/20:4	724.5	583.5	5.74	0.93	17	5	Positive
PE O-36:6	722.5	581.5	5.50	0.70	17	5	Positive
PE O-40:6 | PE P-18:0/22:5	778.5	637.5	5.90	1.20	17	5	Positive
PE P-16:0/18:1 FA	702.5	339.3	6.00	0.70	17	5	Positive
PE P-16:0/18:1 head group	702.5	364.3	6.00	0.70	17	5	Positive
PE P-16:0/18:2 FA	700.5	337.3	5.80	0.70	17	5	Positive
PE P-16:0/18:2 head group	700.5	364.3	5.80	0.70	17	5	Positive
PE P-16:0/20:4 FA	724.5	361.3	5.76	0.70	17	5	Positive
PE P-16:0/20:4 head group	724.5	364.3	5.76	0.70	17	5	Positive
PE P-16:0/22:5 FA	750.5	387.3	5.80	0.70	17	5	Positive
PE P-16:0/22:5 head group	750.5	364.3	5.80	0.70	17	5	Positive
PE P-16:0/22:6 FA	748.5	385.3	5.70	0.70	17	5	Positive
PE P-16:0/22:6 head group	748.5	364.3	5.70	0.70	17	5	Positive
PE P-18:0/18:1 FA	730.5	339.3	6.41	0.70	17	5	Positive
PE P-18:0/18:1 head group	730.5	392.3	6.41	0.70	17	5	Positive
PE P-18:0/18:2 FA	728.6	337.3	6.17	0.70	17	5	Positive
PE P-18:0/18:2 head group	728.6	392.3	6.17	0.70	17	5	Positive
PE P-18:0/20:4 FA	752.6	361.3	6.15	0.70	17	5	Positive
PE P-18:0/20:4 head group	752.6	392.3	6.15	0.70	17	5	Positive
PE P-18:0/22:5 FA	778.5	387.3	6.15	0.70	17	5	Positive
PE P-18:0/22:5 head group	778.5	392.3	6.15	0.70	17	5	Positive
PE P-18:0/22:6 FA	776.6	385.3	6.05	0.70	17	5	Positive
PE P-18:0/22:6 head group	776.6	392.3	6.05	0.70	17	5	Positive
PE P-20:0/20:4 FA	780.6	361.3	6.48	0.70	17	5	Positive
PE P-20:0/20:4 head group	780.6	420.3	6.48	0.70	17	5	Positive
PI 25:0 (IS)	730.3	453.2	3.90	0.70	20	5	Positive
PI 32:0	828.6	551.6	5.07	0.70	17	5	Positive
PI 32:1	826.5	549.5	4.90	0.70	17	5	Positive
PI 34:0	856.6	579.6	5.30	0.70	17	5	Positive
PI 34:1	854.6	577.6	5.30	1.00	17	5	Positive
PI 34:2	852.6	575.6	4.98	1.00	17	5	Positive
PI 36:1	882.6	605.6	5.43	0.70	17	5	Positive
PI 36:2	880.6	603.6	5.40	1.00	17	5	Positive
PI 36:3	878.6	601.6	4.95	1.00	17	5	Positive
PI 36:4	876.6	599.6	4.90	0.70	17	5	Positive
PI 38:2	908.6	631.6	5.40	1.00	17	5	Positive
PI 38:3	906.6	629.6	5.42	0.70	17	5	Positive
PI 38:4	904.6	627.6	5.30	0.70	17	5	Positive
PI 38:5	902.6	625.6	5.02	1.00	17	5	Positive
PI 38:6	900.6	623.6	4.89	0.70	17	5	Positive
PI 40:4	932.6	655.6	5.50	0.70	17	5	Positive
PI 40:5	930.6	653.6	5.40	0.70	17	5	Positive
PI 40:6	928.6	651.6	5.15	0.70	17	5	Positive
PS 28:0 (IS)	680.5	495.5	4.42	1.20	25	5	Positive
PS 34:0	764.5	579.5	5.50	1.00	25	5	Positive
PS 34:1	762.6	577.6	5.23	1.00	25	5	Positive
PS 34:2	760.6	575.6	5.02	1.00	25	5	Positive
PS 36:1	790.6	605.6	5.51	1.00	25	5	Positive
PS 36:2	788.5	603.5	5.29	1.00	25	5	Positive
PS 38:3	814.6	629.6	5.34	1.00	25	5	Positive
PS 38:4	812.5	627.5	5.34	1.00	25	5	Positive
PS 38:5	810.5	625.5	5.09	1.00	25	5	Positive
PS 40:5	838.6	653.6	5.34	1.00	25	5	Positive
PS 40:6	836.5	651.5	5.29	1.00	25	5	Positive

Supplementary Table S11: Transition list for the sphingolipid method.

Compound Name	Precursor m/z	Product m/z	Ret. Time (min)	Delta Ret Time (min)	Collision Energy	Cell Accelerator Voltage	Polarity
Cer d16:1/16:0	510.5	236.2	4.39	1.00	17	5	Positive
Cer d16:1/16:0 (-H2O)	492.5	236.2	4.39	1.00	17	5	Positive
Cer d16:1/18:0	538.5	236.2	4.78	1.00	17	5	Positive
Cer d16:1/18:0 (-H2O)	520.5	236.2	4.78	1.00	17	5	Positive
Cer d16:1/20:0	566.5	236.2	5.12	0.88	17	5	Positive
Cer d16:1/20:0 (-H2O)	548.5	236.2	5.12	0.88	17	5	Positive
Cer d16:1/22:0	594.5	236.2	5.46	0.97	17	5	Positive
Cer d16:1/22:0 (-H2O)	576.5	236.2	5.46	0.97	17	5	Positive
Cer d16:1/22:1	592.5	236.2	5.12	0.97	17	5	Positive
Cer d16:1/22:1 (-H2O)	574.5	236.2	5.12	0.97	17	5	Positive
Cer d16:1/23:0	608.6	236.2	5.62	0.90	17	5	Positive
Cer d16:1/23:0 (-H2O)	590.6	236.2	5.62	0.90	17	5	Positive
Cer d16:1/24:0	622.6	236.2	5.76	0.90	17	5	Positive
Cer d16:1/24:0 (-H2O)	604.6	236.2	5.76	0.90	17	5	Positive
Cer d16:1/24:1	620.6	236.2	5.45	0.97	17	5	Positive
Cer d16:1/24:1 (-H2O)	602.6	236.2	5.45	0.97	17	5	Positive
Cer d16:1/25:1	634.6	236.2	5.65	0.68	17	5	Positive
Cer d16:1/25:1 (-H2O)	616.6	236.2	5.65	0.68	17	5	Positive
Cer d16:1/26:0	650.6	236.2	6.02	0.70	17	5	Positive
Cer d16:1/26:0 (-H2O)	632.6	236.2	6.02	0.70	17	5	Positive
Cer d16:1/26:1	648.6	236.2	5.80	0.91	17	5	Positive
Cer d16:1/26:1 (-H2O)	630.6	236.2	5.80	0.91	17	5	Positive
Cer d18:0/16:0	540.5	266.2	4.89	1.00	17	5	Positive
Cer d18:0/16:0 (-H2O)	522.5	266.2	4.89	1.00	17	5	Positive
Cer d18:0/18:0	568.5	266.2	5.10	1.00	17	5	Positive
Cer d18:0/18:0 (-H2O)	550.5	266.2	5.10	1.00	17	5	Positive
Cer d18:0/20:0	596.5	266.2	5.63	0.64	17	5	Positive
Cer d18:0/20:0 (-H2O)	578.5	266.2	5.63	0.64	17	5	Positive
Cer d18:0/22:0	624.6	266.2	5.83	0.67	17	5	Positive
Cer d18:0/22:0 (-H2O)	606.6	266.2	5.83	0.67	17	5	Positive
Cer d18:0/22:1	622.5	266.2	5.65	0.67	17	5	Positive
Cer d18:0/22:1 (-H2O)	604.5	266.2	5.65	0.67	17	5	Positive
Cer d18:0/23:0	638.6	266.2	5.95	0.90	17	5	Positive
Cer d18:0/23:0 (-H2O)	620.6	266.2	5.95	0.90	17	5	Positive
Cer d18:0/23:1	636.6	266.2	5.99	0.80	17	5	Positive
Cer d18:0/23:1 (-H2O)	618.6	266.2	5.99	0.80	17	5	Positive
Cer d18:0/24:0	652.6	266.2	6.17	1.03	17	5	Positive
Cer d18:0/24:0 (-H2O)	634.6	266.2	6.17	1.03	17	5	Positive
Cer d18:0/24:1	650.6	266.2	5.91	0.90	17	5	Positive
Cer d18:0/24:1 (-H2O)	632.6	266.2	5.91	0.90	17	5	Positive
Cer d18:0/25:0	666.6	266.2	6.29	1.02	17	5	Positive
Cer d18:0/25:0 (-H2O)	648.6	266.2	6.29	1.02	17	5	Positive
Cer d18:0/25:1	664.6	266.2	6.00	1.04	17	5	Positive
Cer d18:0/25:1 (-H2O)	646.6	266.2	6.00	1.04	17	5	Positive
Cer d18:0/26:0	680.6	266.2	6.45	0.70	17	5	Positive
Cer d18:0/26:0 (-H2O)	662.6	266.2	6.45	0.70	17	5	Positive
Cer d18:0/26:1	678.6	266.2	6.21	0.70	17	5	Positive
Cer d18:0/26:1 (-H2O)	660.6	266.2	6.21	0.70	17	5	Positive
Cer d18:1/14:0	510.5	264.2	4.46	1.00	17	5	Positive
Cer d18:1/14:0 (-H2O)	492.5	264.2	4.46	1.00	17	5	Positive
Cer d18:1/14:1	508.5	264.2	4.20	1.00	17	5	Positive
Cer d18:1/14:1 (-H2O)	490.5	264.2	4.20	1.00	17	5	Positive
Cer d18:1/16:0	538.5	264.2	4.76	0.85	17	5	Positive
Cer d18:1/16:0 (-H2O)	520.5	264.2	4.76	0.85	17	5	Positive
Cer d18:1/16:1	536.5	264.2	4.59	0.85	17	5	Positive
Cer d18:1/16:1 (-H2O)	518.5	264.2	4.59	0.85	17	5	Positive
Cer d18:1/17:0 (-H2O) (IS)	534.3	264.3	4.90	1.00	37	5	Positive
Cer d18:1/17:0 (IS)	552.3	264.3	4.90	1.00	37	5	Positive
Cer d18:1/18:0	566.5	264.2	5.12	1.03	17	5	Positive
Cer d18:1/18:0 (-H2O)	548.5	264.2	5.12	1.03	17	5	Positive
Cer d18:1/18:1	564.5	264.2	4.90	1.03	17	5	Positive
Cer d18:1/18:1 (-H2O)	546.5	264.2	4.90	1.03	17	5	Positive
Cer d18:1/20:0	594.5	264.2	5.44	0.97	17	5	Positive
Cer d18:1/20:0 (-H2O)	576.5	264.2	5.44	0.97	17	5	Positive
Cer d18:1/20:1	592.5	264.2	5.26	0.97	17	5	Positive
Cer d18:1/20:1 (-H2O)	574.5	264.2	5.26	0.97	17	5	Positive
Cer d18:1/22:0	622.6	264.2	5.74	0.90	17	5	Positive
Cer d18:1/22:0 (-H2O)	604.6	264.2	5.74	0.90	17	5	Positive
Cer d18:1/22:1	620.6	264.2	5.57	1.01	17	5	Positive
Cer d18:1/22:1 (-H2O)	602.6	264.2	5.57	1.01	17	5	Positive
Cer d18:1/23:0	636.6	264.2	5.88	1.03	17	5	Positive
Cer d18:1/23:0 (-H2O)	618.6	264.2	5.88	1.03	17	5	Positive
Cer d18:1/23:1	634.6	264.2	5.67	0.96	17	5	Positive
Cer d18:1/23:1 (-H2O)	616.6	264.2	5.67	0.96	17	5	Positive
Cer d18:1/24:0	650.6	264.2	6.00	0.90	17	5	Positive
Cer d18:1/24:0 (-H2O)	632.6	264.2	6.00	0.90	17	5	Positive
Cer d18:1/24:1	648.6	264.2	5.73	0.90	17	5	Positive
Cer d18:1/24:1 (-H2O)	630.6	264.2	5.73	0.90	17	5	Positive
Cer d18:1/25:0	664.6	264.2	6.12	0.90	17	5	Positive
Cer d18:1/25:0 (-H2O)	646.6	264.2	6.12	0.90	17	5	Positive
Cer d18:1/25:1	662.6	264.2	5.95	0.90	17	5	Positive
Cer d18:1/25:1 (-H2O)	644.6	264.2	5.95	0.90	17	5	Positive
Cer d18:1/26:0	678.6	264.2	6.24	0.90	17	5	Positive

Compound Name	Precursor m/z	Product m/z	Ret. Time (min)	Delta Ret Time (min)	Collision Energy	Cell Accelerator Voltage	Polarity
Cer d18:1/26:0 (-H2O)	660.6	264.2	6.24	0.90	17	5	Positive
Cer d18:1/26:1	676.6	264.2	6.00	0.91	17	5	Positive
Cer d18:1/26:1 (-H2O)	658.6	264.2	6.00	0.91	17	5	Positive
Cer d18:1/8:0	426.4	264.2	3.26	0.72	17	5	Positive
Cer d18:1/8:0 (-H2O)	408.4	264.2	3.26	0.72	17	5	Positive
Cer d18:2/14:0	508.5	262.2	4.00	1.00	17	5	Positive
Cer d18:2/14:0 (-H2O)	490.5	262.2	4.00	1.00	17	5	Positive
Cer d18:2/16:0	536.5	262.2	4.45	0.68	17	5	Positive
Cer d18:2/16:0 (-H2O)	518.5	262.2	4.45	0.68	17	5	Positive
Cer d18:2/18:0	564.5	262.2	4.86	0.58	17	5	Positive
Cer d18:2/18:0 (-H2O)	546.5	262.2	4.86	0.58	17	5	Positive
Cer d18:2/18:1	562.5	262.2	4.60	1.00	17	5	Positive
Cer d18:2/18:1 (-H2O)	544.5	262.2	4.60	1.00	17	5	Positive
Cer d18:2/20:0	592.5	262.2	5.25	0.91	17	5	Positive
Cer d18:2/20:0 (-H2O)	574.5	262.2	5.25	0.91	17	5	Positive
Cer d18:2/20:1	590.5	262.2	5.05	0.91	17	5	Positive
Cer d18:2/20:1 (-H2O)	572.5	262.2	5.05	0.91	17	5	Positive
Cer d18:2/22:0	620.6	262.2	5.50	0.90	17	5	Positive
Cer d18:2/22:0 (-H2O)	602.6	262.2	5.50	0.90	17	5	Positive
Cer d18:2/22:1	618.6	262.2	5.25	0.90	17	5	Positive
Cer d18:2/22:1 (-H2O)	600.6	262.2	5.25	0.90	17	5	Positive
Cer d18:2/23:0	634.6	262.2	5.66	0.90	17	5	Positive
Cer d18:2/23:0 (-H2O)	616.6	262.2	5.66	0.90	17	5	Positive
Cer d18:2/23:1	632.6	262.2	5.40	0.90	17	5	Positive
Cer d18:2/23:1 (-H2O)	614.6	262.2	5.40	0.90	17	5	Positive
Cer d18:2/24:0	648.6	262.2	5.81	1.08	17	5	Positive
Cer d18:2/24:0 (-H2O)	630.6	262.2	5.81	1.08	17	5	Positive
Cer d18:2/24:1	646.6	262.2	5.60	1.03	17	5	Positive
Cer d18:2/24:1 (-H2O)	628.6	262.2	5.60	1.03	17	5	Positive
Cer d18:2/25:0	662.6	262.2	5.92	0.94	17	5	Positive
Cer d18:2/25:0 (-H2O)	644.6	262.2	5.92	0.94	17	5	Positive
Cer d18:2/25:1	660.6	262.2	5.60	0.74	17	5	Positive
Cer d18:2/25:1 (-H2O)	642.6	262.2	5.60	0.74	17	5	Positive
Cer d18:2/26:0	676.6	262.2	6.00	0.70	17	5	Positive
Cer d18:2/26:0 (-H2O)	658.6	262.2	6.00	0.70	17	5	Positive
Cer d18:2/26:1	674.6	262.2	5.75	0.80	17	5	Positive
Cer d18:2/26:1 (-H2O)	656.6	262.2	5.75	0.80	17	5	Positive
Hex2Cer d16:1/16:0	834.7	236.2	3.79	0.84	45	5	Positive
Hex2Cer d16:1/16:0 (-H2O)	816.7	236.2	3.79	0.84	45	5	Positive
Hex2Cer d18:1/14:0	834.7	264.2	3.78	0.55	45	5	Positive
Hex2Cer d18:1/14:0 (-H2O)	816.7	264.2	3.78	0.55	45	5	Positive
Hex2Cer d18:1/16:0	862.7	264.2	4.17	0.76	45	5	Positive
Hex2Cer d18:1/16:0 (-H2O)	844.7	264.2	4.17	0.76	45	5	Positive
Hex2Cer d18:1/16:0 d3 (IS)	856.6	264.3	4.10	1.00	45	5	Positive
Hex2Cer d18:1/16:0 d3(-H2O) (IS)	847.6	264.3	4.10	1.00	45	5	Positive
Hex2Cer d18:1/18:0	890.7	264.2	4.52	0.55	45	5	Positive
Hex2Cer d18:1/18:0 (-H2O)	872.7	264.2	4.52	0.55	45	5	Positive
Hex2Cer d18:1/20:0	918.7	264.2	4.85	0.59	45	5	Positive
Hex2Cer d18:1/20:0 (-H2O)	900.7	264.2	4.85	0.59	45	5	Positive
Hex2Cer d18:1/22:0	946.7	264.2	5.20	0.73	45	5	Positive
Hex2Cer d18:1/22:0 (-H2O)	928.7	264.2	5.20	0.73	45	5	Positive
Hex2Cer d18:1/22:1	944.7	264.2	4.90	0.73	45	5	Positive
Hex2Cer d18:1/22:1 (-H2O)	926.7	264.2	4.90	0.73	45	5	Positive
Hex2Cer d18:1/23:0	960.7	264.2	5.34	0.63	45	5	Positive
Hex2Cer d18:1/23:0 (-H2O)	942.7	264.2	5.34	0.63	45	5	Positive
Hex2Cer d18:1/24:0	974.7	264.2	5.47	0.80	45	5	Positive
Hex2Cer d18:1/24:0 (-H2O)	956.7	264.2	5.47	0.80	45	5	Positive
Hex2Cer d18:1/24:1	972.7	264.2	5.18	0.91	45	5	Positive
Hex2Cer d18:1/24:1 (-H2O)	954.7	264.2	5.18	0.91	45	5	Positive
Hex2Cer d18:2/16:0	860.7	262.2	3.86	0.91	45	5	Positive
Hex2Cer d18:2/16:0 (-H2O)	842.7	262.2	3.86	0.91	45	5	Positive
Hex2Cer d18:2/18:1	886.7	262.2	4.00	1.00	45	5	Positive
Hex2Cer d18:2/18:1 (-H2O)	868.7	262.2	4.00	1.00	45	5	Positive
Hex2Cer d18:2/22:0	944.7	262.2	4.93	1.00	45	5	Positive
Hex2Cer d18:2/22:0 (-H2O)	926.7	262.2	4.93	1.00	45	5	Positive
Hex2Cer d18:2/24:0	972.7	262.2	5.24	0.80	45	5	Positive
Hex2Cer d18:2/24:0 (-H2O)	954.7	262.2	5.24	0.80	45	5	Positive
Hex2Cer d18:2/24:1	970.7	262.2	4.93	0.80	45	5	Positive
Hex2Cer d18:2/24:1 (-H2O)	952.7	262.2	4.93	0.80	45	5	Positive
GM3 d16:1/14:0	1097.7	236.2	2.98	0.51	55	5	Positive
GM3 d16:1/14:0	1095.7	290.2	2.98	0.51	55	5	Negative
GM3 d16:1/16:0	1125.7	236.2	3.29	0.51	55	5	Positive
GM3 d16:1/16:0	1123.7	290.2	3.29	0.51	55	5	Negative
GM3 d16:1/18:0	1153.7	236.2	3.64	0.78	55	5	Positive
GM3 d16:1/18:0	1151.7	290.2	3.64	0.78	55	5	Negative
GM3 d16:1/20:0	1181.8	236.2	3.98	0.59	55	5	Positive
GM3 d16:1/20:0	1179.8	290.2	3.98	0.59	55	5	Negative
GM3 d16:1/22:0	1209.8	236.2	4.32	0.59	55	5	Positive
GM3 d16:1/22:0	1207.8	290.2	4.32	0.59	55	5	Negative
GM3 d16:1/24:0	1237.8	236.2	4.64	0.59	55	5	Positive
GM3 d16:1/24:0	1235.8	290.2	4.64	0.59	55	5	Negative
GM3 d16:1/24:1	1235.8	236.2	4.40	0.59	55	5	Positive
GM3 d16:1/24:1	1233.8	290.2	4.40	0.59	55	5	Negative
GM3 d18:1/14:0	1125.7	264.2	3.30	0.70	55	5	Positive
GM3 d18:1/14:0	1123.7	290.2	3.30	0.70	55	5	Negative
GM3 d18:1/16:0	1153.7	264.2	3.60	1.02	55	5	Positive

Compound Name	Precursor m/z	Product m/z	Ret. Time (min)	Delta Ret Time (min)	Collision Energy	Cell Accelerator Voltage	Polarity
GM3 d18:1/16:0	1151.7	290.2	3.60	1.02	55	5	Negative
GM3 d18:1/18:0	1181.8	264.2	3.98	0.74	55	5	Positive
GM3 d18:1/18:0	1179.8	290.2	3.98	0.74	55	5	Negative
GM3 d18:1/20:0	1209.8	264.2	4.36	0.74	55	5	Positive
GM3 d18:1/20:0	1207.8	290.2	4.36	0.74	55	5	Negative
GM3 d18:1/22:0	1237.8	264.2	4.64	0.59	55	5	Positive
GM3 d18:1/22:0	1235.8	290.2	4.64	0.59	55	5	Negative
GM3 d18:1/24:0	1265.8	264.2	4.94	1.02	55	5	Positive
GM3 d18:1/24:0	1263.8	290.2	4.94	1.02	55	5	Negative
GM3 d18:1/24:1	1263.8	264.2	4.65	0.86	55	5	Positive
GM3 d18:1/24:1	1261.8	290.2	4.65	0.86	55	5	Negative
GM3 d18:2/14:0	1123.7	262.2	3.00	0.70	55	5	Positive
GM3 d18:2/14:0	1121.7	290.2	3.00	0.70	55	5	Negative
GM3 d18:2/16:0	1151.7	262.2	3.38	0.70	55	5	Positive
GM3 d18:2/16:0	1149.7	290.2	3.38	0.70	55	5	Negative
GM3 d18:2/18:0	1179.8	262.2	3.71	0.82	55	5	Positive
GM3 d18:2/18:0	1177.8	290.2	3.71	0.82	55	5	Negative
GM3 d18:2/20:0	1207.8	262.2	4.06	0.67	55	5	Positive
GM3 d18:2/20:0	1205.8	290.2	4.06	0.67	55	5	Negative
GM3 d18:2/22:0	1235.8	262.2	4.40	0.78	55	5	Positive
GM3 d18:2/22:0	1233.8	290.2	4.40	0.78	55	5	Negative
GM3 d18:2/24:0	1263.8	262.2	4.63	0.98	55	5	Positive
GM3 d18:2/24:0	1261.8	290.2	4.63	0.98	55	5	Negative
GM3 d18:2/24:1	1261.8	262.2	4.41	0.74	55	5	Positive
GM3 d18:2/24:1	1259.8	290.2	4.41	0.74	55	5	Negative
HexCer d16:1/16:0	672.6	236.2	3.99	0.67	30	5	Positive
HexCer d16:1/16:0 (-H2O)	654.6	236.2	3.99	0.67	30	5	Positive
HexCer d16:1/20:0	728.6	236.2	4.75	0.57	30	5	Positive
HexCer d16:1/20:0 (-H2O)	710.6	236.2	4.75	0.57	30	5	Positive
HexCer d16:1/22:0	756.6	236.2	5.08	0.67	30	5	Positive
HexCer d16:1/22:0 (-H2O)	738.6	236.2	5.08	0.67	30	5	Positive
HexCer d16:1/22:1	754.6	236.2	4.98	0.67	30	5	Positive
HexCer d16:1/22:1 (-H2O)	736.6	236.2	4.98	0.67	30	5	Positive
HexCer d16:1/23:0	770.6	236.2	5.26	0.62	30	5	Positive
HexCer d16:1/23:0 (-H2O)	752.6	236.2	5.26	0.62	30	5	Positive
HexCer d16:1/23:1	768.6	236.2	5.00	1.00	30	5	Positive
HexCer d16:1/23:1 (-H2O)	750.6	236.2	5.00	1.00	30	5	Positive
HexCer d16:1/24:0	784.6	236.2	5.40	0.64	30	5	Positive
HexCer d16:1/24:0 (-H2O)	766.6	236.2	5.40	0.64	30	5	Positive
HexCer d16:1/24:1	782.6	236.2	5.07	0.68	30	5	Positive
HexCer d16:1/24:1 (-H2O)	764.6	236.2	5.07	0.68	30	5	Positive
HexCer d16:1/26:0	812.7	236.2	5.60	1.00	30	5	Positive
HexCer d16:1/26:0 (-H2O)	794.7	236.2	5.60	1.00	30	5	Positive
HexCer d18:0/16:0	702.6	266.2	4.40	1.00	30	5	Positive
HexCer d18:0/16:0 (-H2O)	684.6	266.2	4.40	1.00	30	5	Positive
HexCer d18:0/16:1	700.6	266.2	4.65	0.68	30	5	Positive
HexCer d18:0/16:1 (-H2O)	682.6	266.2	4.65	0.68	30	5	Positive
HexCer d18:0/22:0	786.6	266.2	5.36	0.63	30	5	Positive
HexCer d18:0/22:0 (-H2O)	768.6	266.2	5.36	0.63	30	5	Positive
HexCer d18:0/22:1	784.6	266.2	5.27	0.63	30	5	Positive
HexCer d18:0/22:1 (-H2O)	766.6	266.2	5.27	0.63	30	5	Positive
HexCer d18:0/23:0	800.7	266.2	5.52	0.66	30	5	Positive
HexCer d18:0/23:0 (-H2O)	782.7	266.2	5.52	0.66	30	5	Positive
HexCer d18:0/24:0	814.7	266.2	5.65	0.66	30	5	Positive
HexCer d18:0/24:0 (-H2O)	796.7	266.2	5.65	0.66	30	5	Positive
HexCer d18:0/24:1	812.7	266.2	5.37	0.74	30	5	Positive
HexCer d18:0/24:1 (-H2O)	794.7	266.2	5.37	0.74	30	5	Positive
HexCer d18:1/14:0	672.6	264.2	3.95	1.00	30	5	Positive
HexCer d18:1/14:0 (-H2O)	654.6	264.2	3.95	1.00	30	5	Positive
HexCer d18:1/16:0	700.6	264.2	4.36	0.96	30	5	Positive
HexCer d18:1/16:0 (-H2O)	682.6	264.2	4.36	0.96	30	5	Positive
HexCer d18:1/16:0 d3 (-H2O) (IS)	685.6	264.3	4.28	0.70	37	5	Positive
HexCer d18:1/16:0 d3 (IS)	703.6	264.3	4.28	0.70	37	5	Positive
HexCer d18:1/16:1	698.6	264.2	4.24	0.96	30	5	Positive
HexCer d18:1/16:1 (-H2O)	680.6	264.2	4.24	0.96	30	5	Positive
HexCer d18:1/18:0	728.6	264.2	4.72	0.80	30	5	Positive
HexCer d18:1/18:0 (-H2O)	710.6	264.2	4.72	0.80	30	5	Positive
HexCer d18:1/18:1	726.6	264.2	4.43	0.80	30	5	Positive
HexCer d18:1/18:1 (-H2O)	708.6	264.2	4.43	0.80	30	5	Positive
HexCer d18:1/20:0	756.6	264.2	5.06	0.72	30	5	Positive
HexCer d18:1/20:0 (-H2O)	738.6	264.2	5.06	0.72	30	5	Positive
HexCer d18:1/20:1	754.6	264.2	4.95	0.72	30	5	Positive
HexCer d18:1/20:1 (-H2O)	736.6	264.2	4.95	0.72	30	5	Positive
HexCer d18:1/22:0	784.6	264.2	5.36	0.90	30	5	Positive
HexCer d18:1/22:0 (-H2O)	766.6	264.2	5.36	0.90	30	5	Positive
HexCer d18:1/22:1	782.6	264.2	5.28	1.02	30	5	Positive
HexCer d18:1/22:1 (-H2O)	764.6	264.2	5.28	1.02	30	5	Positive
HexCer d18:1/23:0	798.6	264.2	5.50	0.85	30	5	Positive
HexCer d18:1/23:0 (-H2O)	780.6	264.2	5.50	0.85	30	5	Positive
HexCer d18:1/23:1	796.6	264.2	5.45	1.00	30	5	Positive
HexCer d18:1/23:1 (-H2O)	778.6	264.2	5.45	1.00	30	5	Positive
HexCer d18:1/24:0	812.7	264.2	5.64	0.90	30	5	Positive
HexCer d18:1/24:0 (-H2O)	794.7	264.2	5.64	0.90	30	5	Positive
HexCer d18:1/24:1	810.7	264.2	5.36	0.90	30	5	Positive
HexCer d18:1/24:1 (-H2O)	792.7	264.2	5.36	0.90	30	5	Positive
HexCer d18:1/25:0	826.7	264.2	5.80	1.00	30	5	Positive

Compound Name	Precursor m/z	Product m/z	Ret. Time (min)	Delta Ret Time (min)	Collision Energy	Cell Accelerator Voltage	Polarity
HexCer d18:1/25:0 (-H2O)	808.7	264.2	5.80	1.00	30	5	Positive
HexCer d18:1/25:1	824.7	264.2	5.52	0.74	30	5	Positive
HexCer d18:1/25:1 (-H2O)	806.7	264.2	5.52	0.74	30	5	Positive
HexCer d18:1/26:0	840.7	264.2	5.91	0.69	30	5	Positive
HexCer d18:1/26:0 (-H2O)	822.7	264.2	5.91	0.69	30	5	Positive
HexCer d18:1/26:1	838.7	264.2	5.65	1.00	30	5	Positive
HexCer d18:1/26:1 (-H2O)	820.7	264.2	5.65	1.00	30	5	Positive
HexCer d18:2/16:0	698.6	262.2	4.04	0.61	30	5	Positive
HexCer d18:2/16:0 (-H2O)	680.6	262.2	4.04	0.61	30	5	Positive
HexCer d18:2/16:1	696.6	262.2	3.93	1.00	30	5	Positive
HexCer d18:2/16:1 (-H2O)	678.6	262.2	3.93	1.00	30	5	Positive
HexCer d18:2/20:0	754.6	262.2	4.80	0.62	30	5	Positive
HexCer d18:2/20:0 (-H2O)	736.6	262.2	4.80	0.62	30	5	Positive
HexCer d18:2/22:0	782.6	262.2	5.14	0.80	30	5	Positive
HexCer d18:2/22:0 (-H2O)	764.6	262.2	5.14	0.80	30	5	Positive
HexCer d18:2/22:1	780.6	262.2	5.04	0.80	30	5	Positive
HexCer d18:2/22:1 (-H2O)	762.6	262.2	5.04	0.80	30	5	Positive
HexCer d18:2/23:0	796.6	262.2	5.29	0.73	30	5	Positive
HexCer d18:2/23:0 (-H2O)	778.6	262.2	5.29	0.73	30	5	Positive
HexCer d18:2/24:0	810.7	262.2	5.44	0.74	30	5	Positive
HexCer d18:2/24:0 (-H2O)	792.7	262.2	5.44	0.74	30	5	Positive
HexCer d18:2/24:1	808.7	262.2	5.14	0.68	30	5	Positive
HexCer d18:2/24:1 (-H2O)	790.7	262.2	5.14	0.68	30	5	Positive
HexCer d18:2/25:0	824.7	262.2	5.62	0.68	30	5	Positive
HexCer d18:2/25:0 (-H2O)	806.7	262.2	5.62	0.68	30	5	Positive
SM C30:1 (IS)	647.6	184.1	3.39	1.00	25	5	Positive
SM C32:1	675.5	184.1	3.77	1.00	25	5	Positive
SM C32:2	673.5	184.1	3.46	1.00	25	5	Positive
SM C34:0	705.6	184.1	4.16	1.00	25	5	Positive
SM C34:1	703.6	184.1	4.17	1.00	25	5	Positive
SM C34:2	701.6	184.1	3.86	1.00	25	5	Positive
SM C36:0	733.6	184.1	4.50	0.80	25	5	Positive
SM C36:1	731.6	184.1	4.52	1.00	25	5	Positive
SM C36:2	729.6	184.1	4.25	1.00	25	5	Positive
SM C38:0	761.6	184.1	4.60	1.00	25	5	Positive
SM C38:1	759.6	184.1	4.88	1.00	25	5	Positive
SM C38:2	757.6	184.1	4.62	1.00	25	5	Positive
SM C39:1	773.6	184.1	5.09	1.00	25	5	Positive
SM C40:0	789.7	184.1	4.90	1.00	25	5	Positive
SM C40:1	787.7	184.1	5.25	1.00	25	5	Positive
SM C40:2	785.7	184.1	4.95	1.00	25	5	Positive
SM C41:1	801.7	184.1	5.40	1.00	25	5	Positive
SM C41:2	799.7	184.1	5.14	1.00	25	5	Positive
SM C42:0	817.7	184.1	5.64	1.00	25	5	Positive
SM C42:1	815.7	184.1	5.50	1.00	25	5	Positive
SM C42:2	813.7	184.1	5.23	1.00	25	5	Positive
SM C42:3	811.7	184.1	4.99	1.00	25	5	Positive
SM C43:1	829.7	184.1	5.60	1.00	25	5	Positive
SM C43:2	827.7	184.1	5.34	1.00	25	5	Positive
SM C44:1	843.7	184.1	5.99	1.00	25	5	Positive
SM d16:1/16:0	675.5	236.2	3.77	0.80	37	5	Positive
SM d16:1/18:0	703.6	236.2	4.16	0.80	37	5	Positive
SM d16:1/20:0	731.6	236.2	4.53	0.80	37	5	Positive
SM d16:1/22:0	759.6	236.2	4.92	0.80	37	5	Positive
SM d16:1/23:0	773.6	236.2	5.09	0.80	37	5	Positive
SM d16:1/24:0	787.7	236.2	5.26	0.80	37	5	Positive
SM d16:1/24:1	785.7	236.2	4.95	0.80	37	5	Positive
SM d18:0/16:0	705.6	266.2	4.16	0.80	37	5	Positive
SM d18:0/18:0	733.6	266.2	4.50	0.80	37	5	Positive
SM d18:0/20:0	761.6	266.2	4.61	0.80	37	5	Positive
SM d18:0/22:0	789.7	266.2	4.94	0.80	37	5	Positive
SM d18:0/24:0	817.7	266.2	5.54	0.80	37	5	Positive
SM d18:1/12:0	647.6	264.2	3.39	0.80	37	5	Positive
SM d18:1/14:0	675.5	264.2	3.77	0.80	37	5	Positive
SM d18:1/16:0	703.6	264.2	4.15	0.80	37	5	Positive
SM d18:1/18:0	731.6	264.2	4.52	0.80	37	5	Positive
SM d18:1/20:0	759.6	264.2	4.89	0.80	37	5	Positive
SM d18:1/22:0	787.7	264.2	5.23	0.80	37	5	Positive
SM d18:1/22:1	785.7	264.2	4.95	0.80	37	5	Positive
SM d18:1/23:0	801.7	264.2	5.40	0.80	37	5	Positive
SM d18:1/23:1	799.7	264.2	5.07	0.80	37	5	Positive
SM d18:1/24:0	815.7	264.2	5.53	0.80	37	5	Positive
SM d18:1/24:1	813.7	264.2	5.23	0.80	37	5	Positive
SM d18:1/25:0	829.7	264.2	5.60	0.80	37	5	Positive
SM d18:1/25:1	827.7	264.2	5.53	0.80	37	5	Positive
SM d18:1/26:0	843.7	264.2	5.99	0.80	37	5	Positive
SM d18:2/14:0	673.5	262.2	3.46	0.80	37	5	Positive
SM d18:2/16:0	701.6	262.2	3.86	0.80	37	5	Positive
SM d18:2/18:0	729.6	262.2	4.25	0.80	37	5	Positive
SM d18:2/20:0	757.6	262.2	4.62	0.80	37	5	Positive
SM d18:2/22:0	785.7	262.2	4.99	0.80	37	5	Positive
SM d18:2/23:0	799.7	262.2	5.15	0.80	37	5	Positive
SM d18:2/24:0	813.7	262.2	5.30	0.80	37	5	Positive
SM d18:2/24:1	811.7	262.2	4.99	0.84	37	5	Positive
SM d18:2/25:0	827.7	262.2	5.50	0.80	37	5	Positive

Supplementary Table S12: Transition list for the triacylglycerol method.

Lipid Species	Q3 Mass (Da)	Dwell time(msec)
d5-TAG 16:0/16:0/16:0 (ISTD)	829.8	10
TAG 10:0/10:0/10:0 (STD)	572.5	10
TAG 12:0/12:0/12:0 (STD)	656.6	10
TAG 14:0/14:0/14:0 (STD)	740.7	10
TAG 46:0	796.7	10
TAG 46:1	794.7	10
TAG 46:2	792.7	10
TAG 48:0	824.8	10
TAG 48:1	822.8	10
TAG 48:2	820.8	10
TAG 48:3	818.8	10
TAG 49:1	836.8	10
TAG 50:0	852.8	10
TAG 50:1	850.8	10
TAG 50:2	848.8	10
TAG 50:3	846.8	10
TAG 50:4	844.8	10
TAG 51:0	866.8	10
TAG 51:1	864.8	10
TAG 51:2	862.8	10
TAG 51:3	860.8	10
TAG 52:1	878.8	10
TAG 52:2	876.8	10
TAG 52:3	874.8	10
TAG 52:4	872.8	10
TAG 52:5	870.8	10
TAG 53:2	890.8	10
TAG 54:0	908.8	10
TAG 54:1	906.8	10
TAG 54:2	904.8	10
TAG 54:3	902.8	10
TAG 54:4	900.8	10
TAG 54:5	898.8	10
TAG 54:6	896.8	10
TAG 55:0	922.8	10
TAG 55:1	920.8	10
TAG 56:5	926.8	10
TAG 56:6	924.8	10
TAG 57:1	948.8	10
TAG 57:2	946.8	10
TAG 57:3	944.8	10
TAG 57:4	942.8	10

Supplementary Table S13: Transition list for the sphingosine 1-phosphate method.

Compound Name	Precursor m/z	Product m/z	Collision Energy	Cell Accelerator Voltage	Polarity
S1P d18:1-13C2D2 (IS)	440.3	60.1	29	3	Positive
S1P d18:1-13C2D2 (IS)	440.3	113	29	3	Positive
S1P d16:1	408.3	60.1	29	3	Positive
S1P d16:1	408.3	113	29	3	Positive
S1P d17:1	422.3	60.1	29	3	Positive
S1P d17:1	422.3	113	29	3	Positive
S1P d18:0	438.3	60.1	29	3	Positive
S1P d18:0	438.3	113	29	3	Positive
S1P d18:1	436.3	60.1	29	3	Positive
S1P d18:1	436.3	113	29	3	Positive
S1P d18:2	434.3	60.1	29	3	Positive
S1P d18:2	434.3	113	29	3	Positive
S1P d19:1	450.4	60.1	29	3	Positive
S1P d19:1	450.4	113	29	3	Positive

Supplementary Table S14: Normalization using Internal Standards (ISTD). The internal standard indicated in the second column was used to normalize all species from the corresponding lipid class indicated in the first column. * indicates that an ISTD from another lipid class (which may have a different response factor) was used for normalization and therefore the calculated abundance will deviate from actual abundance. # indicates that normalization by this ISTD lead to an increase in Coefficient of Variation (CV) for all species of the corresponding class, and therefore the average peak area of the corresponding ISTD in all samples was used to normalize all species of the corresponding class and to calculate the abundances.

Lipid Class	Normalized with Internal Standard
**CE**	PC 28:0 (IS) *
**Cer**	Cer d18:1/17:0 (IS) #
**DG**	DG 12:0_12:0 (IS) #
**Hex1Cer**	Hex1Cer d18:1/16:0 d3 (IS)
**Hex2Cer**	Hex2Cer d18:1/16:0 d3 (IS)
**GM3**	Hex2Cer d18:1/16:0 d3 (IS) *
**LPC**	LPC 20:0 (IS)
**LPC-O**	LPC 20:0 (IS) *
**LPE**	LPE 14:0 (IS)
**PC**	PC 28:0 (IS)
**PC-O**	PC 28:0 (IS) *
**PC-P**	PC 28:0 (IS) *
**PE**	PE 28:0 (IS)
**PE-O**	PE 28:0 (IS) *
**PE-P**	PE 28:0 (IS) *
**PI**	PI 25:0 (IS)
**PS**	PS 28:0 (IS)
**S1P**	S1P d18:1-13C2D2 (IS)
**SM**	SM 30:1 (IS)
**TAG**	d5 TAG 48:0 (IS)
